# Rethinking energy transition strategies for the European Union amid rising energy prices

**DOI:** 10.1073/pnas.2609606123

**Published:** 2026-06-01

**Authors:** Wenjun Meng, Jaime Nieto, Dabo Guan, Jing Meng, Robert Sander, Ulrich Pöschl, Klaus Hubacek, Hang Su, Shu Tao, Yafang Cheng

**Affiliations:** ^a^ Aerosol Chemistry Department, Max Planck Institute for Chemistry, Mainz 55128, Germany; ^b^ College of Urban and Environmental Sciences, Peking University, Beijing 100871, China; ^c^ Research Group on Energy, Economy and System Dynamics, Escuela de Ingenierías Industriales, University of Valladolid, Valladolid 47011, Spain; ^d^ Department of Earth System Science, Tsinghua University, Beijing 100084, China; ^e^ The Bartlett School of Sustainable Construction, University College London, London WC1E 7HB, United Kingdom; ^f^ International Institute for Applied Systems Analysis, Laxenburg A-2361, Austria; ^g^ Multiphase Chemistry Department, Max Planck Institute for Chemistry, Mainz 55128, Germany; ^h^ Integrated Research on Energy, Environment and Society, Energy and Sustainability Research Institute Groningen, University of Groningen, Groningen 9747 AG, The Netherlands; ^i^ State Key Laboratory of Atmospheric Environment and Extreme Meteorology, Institute of Atmospheric Physics, Chinese Academy of Sciences, Beijing 100029, China; ^j^ School of Environmental Science and Engineering, Southern University of Science and Technology, Shenzhen 518055, China

**Keywords:** energy price, energy transition, air quality, health benefits, cost analysis

## Abstract

Geopolitical tensions and the disruption of major energy imports have sharply elevated energy prices across the European Union (EU), creating a persistent energy supply gap and posing long-term challenges for energy security. Here, we introduce a comprehensive framework integrating the advanced Greenhouse Gas and Air Pollution Interactions and Synergies (GAINS) model with a system dynamic WILIAM (“Within limits”) Integrated Assessment Model to systematically evaluate strategies for closing this energy gap. We find that measures to close the energy gap in the short term exacerbate either economic or public health burdens, highlighting the need for more sustainable solutions in the long term. Our further analysis shows that long-term strategies focused on renewable energy transitions not only close the energy gap but also bring net benefits equivalent to ~3% of projected GDP for 2050. Moreover, we find that rising energy prices substantially influence the cost-effectiveness of accelerated energy transitions. Under current high energy price levels, the benefits from achieving energy targets set for 2050 by 5 to 20 y in advance could outweigh the additional costs. The consideration of dynamic trends in the levelized cost of electricity (LCOE), particularly under rapid renewables expansion, is critical for avoiding overly optimistic estimations of green transition benefits. Our findings suggest that a timely transition to renewable energy benefits the EU in the face of the energy crisis, and policies proactively adjusting energy prices could facilitate a greener, more sustainable future.

## Significance

The energy crisis triggered by geopolitical tensions and reduced energy imports has exposed the EU’s vulnerability to energy price spikes and supply disruptions. An integrated assessment linking short-term responses with long-term pathways across economy, environment, climate, and health is needed for comprehensive policy guidance. We show that delaying the phase-out of solid fuels such as coal may alleviate immediate energy shortages but can increase economic burdens or public health risks. Promoting the renewable transition in the long term will not only mitigate the negative economic impacts but also yield climate and health benefits. Given rising energy prices, renewables offer more pronounced advantages over fossil fuels. Accelerating the energy transition harnesses these advantages to deliver greater benefits for the EU’s future development.

Since the late 1970s, the European Union (EU) has relied heavily on imported oil and natural gas, primarily from Russia, Norway, and Algeria (1, 2). By 2020, imports constituted approximately two-thirds of EU’s total energy supply (2). Recent geopolitical challenges have pressured the import-oriented energy strategy (3–5), causing energy prices to spike around 2022 (6) and raising concerns about securing energy supplies (7). In response, the EU has announced plans like REPowerEU to reduce Russian imports (8, 9). The consequent energy gap can be narrowed in a short time frame with various potential strategies to boost supply or reduce demand. In view of the current geopolitical situation, the energy gap is expected to have a persistent and long-term impact. Therefore, there is a compelling necessity for long-term sustainable strategies that extend beyond merely solving the immediate crisis. Transitioning from fossil fuels to renewable energy supplies aligns with the EU’s commitment to mitigating global warming (10) and is integral to achieving the goals outlined in the European Green Deal (11), which are underscored by initiatives such as the Net Zero Industry Act (12) and the European Climate Law (13).

However, geopolitical uncertainties and persistent volatility in energy import prices complicate the process of determining the optimal pace and scale of the renewable energy transition. Existing studies have examined specific shocks or isolated events (e.g., the replacement of Russian natural gas or reductions in gas imports) and their short-term impacts (14–18), or longer-term transition questions such as reduced gas dependence, decarbonization, energy security, and electricity market sensitivities (19–21). Yet these studies typically address either short-term disruptions or individual dimensions, rather than how sustained high energy prices influence the transition pathway in the long term. They also rarely jointly integrated economic costs, public health impacts, and climate benefits within a unified assessment framework. Moreover, key determinants of renewable transition costs, including infrastructure investment, end-use equipment replacement, grid expansion, and dynamic constraints such as renewable energy expansion limits and curtailment challenges are often only partially represented; integrating these dynamic factors is crucial for evaluating the cost-effectiveness of various energy transition pathways. Although recent modeling frameworks have incorporated some of the factors (22), a comprehensive analysis on both costs and benefits under high energy price volatility is still lacking (14, 19, 23).

To more accurately assess EU’s energy strategies, we develop an innovative assessment framework combing a system dynamic WILIAM (“Within limits”) Integrated Assessment Model (IAM) and an advanced sector-focused IAM, Greenhouse Gas and Air Pollution Interactions and Synergies (GAINS) model, which allows a robust assessment of their short- and long-term impacts at the national scale. Specifically, we explore different measures to close the energy gap, explicitly considering both supply-side interventions (e.g., increasing renewable energy capacity and electrification) and demand-side actions (e.g., promoting sustainable lifestyles). Our analysis demonstrates that closing the energy gap in the short term through energy demand reductions or substitutions with solid fuels exacerbates either economic or public health burdens, calling for more sustainable strategies in the long term. In particular, we show that long-term renewable energy strategies not only bridge the energy gap effectively but also yield substantial benefits for climate change mitigation and public health improvements, which significantly outweigh the associated costs. We also evaluate the costs and benefits of accelerating EU’s energy transition and find that the rising energy prices have enhanced the economic attractiveness and cost-effectiveness of energy transition acceleration. This is the case even for the much more ambitious net-zero emission targets, where it is important to consider the rise of material and construction costs due to the sudden surge of demand for renewables. Overall, our work highlights the importance and feasibility of accelerating a sustainable and cost-effective renewable energy transition amid ongoing geopolitical and energy market challenges.

## Results

### Energy Supply Gap and Reactive Measures.

Natural gas and oil accounted for 21% and 44% of the EU’s energy supply, with 33% imported from Russia in 2020 (2) (*SI Appendix*, Fig. S1). Banning Russian imports leads to an average 19% energy supply gap, with central and eastern European countries having the largest gap (*SI Appendix*, Fig. S1). In general, measures to close the energy gap fall into two categories: 1) supply-side measures, such as adjusting the energy mix with more solid fuels and 2) demand-side measures, such as promoting lifestyle changes to reduce energy consumption. The recent trends of energy consumption in the EU suggest that both types of measures have been implemented: Energy consumption in the second half of 2022 was consistently much lower than that in 2021, and natural gas consumption fell faster than the other energy types (2). A weather-adjusted analysis also reveals a natural gas consumption reduction attributable to the energy crisis (17).

**Fig. 1.**
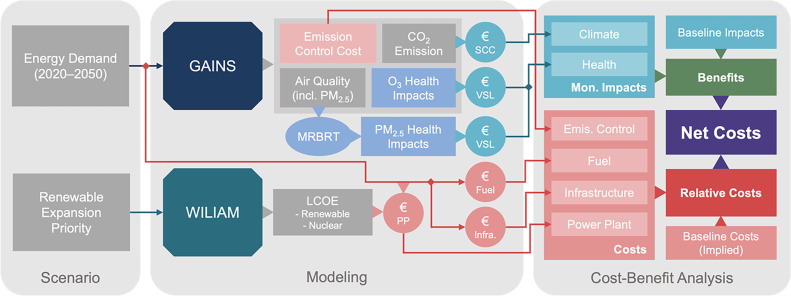
Schematic diagram of the research methodology framework. This framework illustrates the integration of scenario development, modeling, and cost–benefit analysis. Energy demand scenarios and renewable expansion priorities serve as key inputs to two core models: GAINS and WILIAM, respectively. The GAINS model estimates emission control costs, CO_2_ emissions, air quality (PM_2.5_ concentration), and related health impacts from O_3_. The PM_2.5_ concentration is combined with dose–response function Meta-Regression-Bayesian, Regularized, Trimmed splines (MRBRT) to derive the health impacts of PM_2.5_. These results are used to quantify benefits through the social cost of carbon (SCC) and the value of a statistical life (VSL). The WILIAM model assesses the levelized cost of electricity (LCOE) for renewables and nuclear, which informs cost estimates for power plant investments (PP). Scenario-specific energy demand is also used to estimate fuel and infrastructure (Infra.) costs. Outputs from both models feed into the cost–benefit analysis, where monetized climate and health benefits are weighed against various cost components. The net costs in each scenario are determined by comparison with the baseline.

**Fig. 2.**
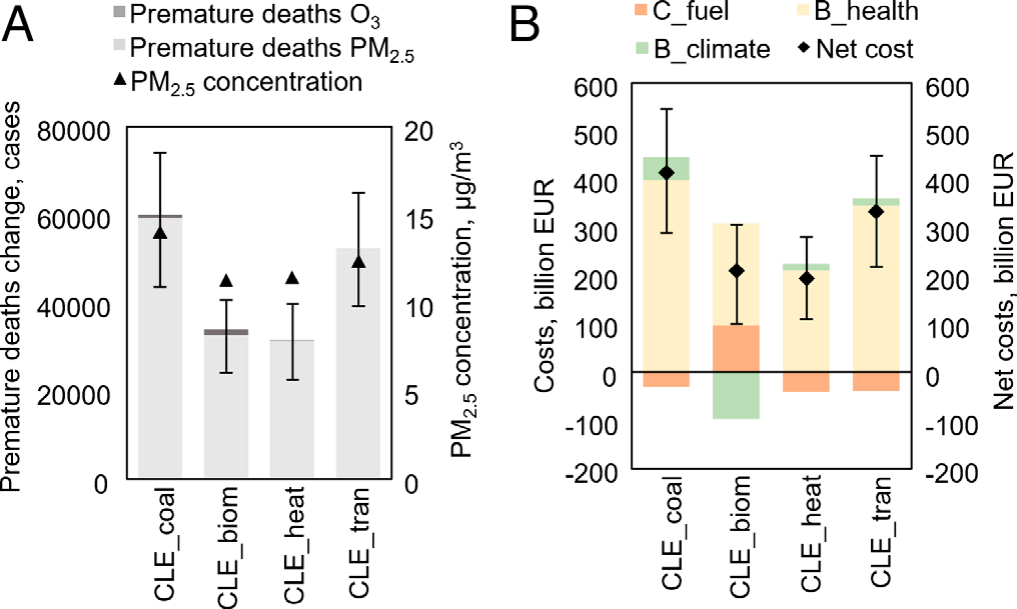
Short-term impacts of measures to close the energy gap in 2025. (*A*) Mean population-weighted PM_2.5_ concentrations (*Right* axis) and associated premature deaths change due to PM_2.5_ and O_3_ exposure (*Left* axis). (*B*) Comparison of total costs and monetized benefits (*Left* axis), and resulting net costs (*Right* axis), relative to the CLE_base scenario. Cost components include fuel costs (C_fuel), monetized health benefits (B_health), and monetized climate benefits (B_climate). Error bars represent 95% CI.

To evaluate impacts of the different measures, we applied the GAINS model to conduct the cost and benefit analysis through the integrated assessment framework (Fig. 1). Fig. 2 shows the annualized costs and benefits of different measures in 2025 (expressed in 2021 EUR). The evaluated measures include supply-side solutions CLE_coal and CLE_biom, representing scenarios where coal or biomass is used to fill the energy gap, respectively; and demand-side solutions CLE_heat and CLE_tran representing scenarios where heating or private transportation demand is reduced, respectively. Here, CLE denotes the current legislation scenario, developed based on the framework of the ECLIPSE (Evaluating the Climate and Air Quality Impacts of Short-Lived Pollutants) V6b (24). It assumes the successful implementation of existing air pollution legislation and incorporates anticipated effects from announced energy, climate, and air quality policies, reflecting established targets and strategic plans (25). We define this CLE scenario as CLE_base in our analysis, serving as the baseline projection of energy and emissions under current policies without additional interventions or policy expansions. From CLE_base, the supply-side scenarios were developed by filling the energy gap with coal or biomass while the demand-side scenarios were developed by reducing heating or transport demand aligning with recommendations by European Commission (26), with hard coal filling the remaining energy gap. More details are described in *Materials and Methods* and *SI Appendix*, Supplementary Note 1.

As shown in Fig. 2, compared to CLE_base, the supply-side measures CLE_coal and CLE_biom would increase the excess premature mortality due to air pollution by 60,000 (44,000 to 75,000) and 34,000 (25,000 to 42,000) cases, corresponding to monetized health costs of 394 (284 to 505) billion EUR and 212 (153 to 271) billion EUR, respectively. While CLE_coal could save 30 (17 to 32) billion EUR for fuel costs due to the lower price of coal, this benefit is insufficient to offset the additional health and climate burden, resulting in a net cost of 414 (288 to 546) billion EUR in 2025. In contrast, CLE_biom does not yield fuel cost savings, as biomass combustion is less efficient and replacing natural gas with biomass results in higher fuel expenditures—an increase of 96 (86 to 107) billion EUR compared to CLE_base. However, because biomass is considered carbon neutral, CLE_biom generates climate benefits by saving 530 Mt CO_2_ in 2025 [98 (62 to 154) billion EUR]. The net cost for CLE_biom is 211 (99 to 305) billion EUR. On the demand side, reducing heating demand by 3 °C (CLE_heat) and cutting private transportation demand by 20% (CLE_tran) only achieve modest reductions of 3% and 4% in total energy demand, respectively. Such reductions are insufficient to close the energy gap, and the remaining energy gaps are filled by increased coal use, resulting in additional health burdens. CLE_heat and CLE_tran scenarios exhibit net costs of 194 (110 to 280) billion EUR and 331 (218 to 448) billion EUR in 2025, respectively. More details are in *SI Appendix*, Supplementary Note 2.

Among the measures analyzed (Fig. 2*B*), CLE_heat results in the lowest additional costs for addressing the energy crisis in the very short term (in 2025). However, it still leads to increased net costs. Also lowering the thermostat is not sustainable, as indoor heating temperature is a key indicator of quality of life. Reducing private transportation and promoting public transportation is a sustainable strategy (27, 28), even though the role of closing the energy gap is limited as shown in our analysis. Hence, our results show that in short term, the measures to close the energy gap increase either the economic or public health burdens, underscoring the need for long-term, sustainable and better-planned strategies in the energy sector.

### Costs and Benefits of Future Strategies.

Recent geopolitical situation indicates that the energy gap from halting Russian imports is likely to persist, and the risks pressurize the EU to explore long-term alternative solutions to close this gap. Similar to short-term impacts, we also examined scenarios from both supply side and demand side for the long-term impacts. By extending the CLE_base, we developed CLE_coal from the supply side and CLE_tran from the demand side for 2050. Note that, for the long-term strategies, we did not analyze CLE_biom and CLE_heat as they are not sustainable for long term because biomass does not yield fuel cost savings and reducing heating demand sacrifices the quality of life. Moreover, since the GAINS model does not consider the investment costs for power plants, we applied the WILIAM to incorporate comprehensive factors such as flexibility management options, curtailment, and CO_2_ prices (29, 30), to reflect the trends of LCOE for renewable energy. Details are provided in *Materials and Methods* and *SI Appendix*, Supplementary Note 1.

**Fig. 3.**
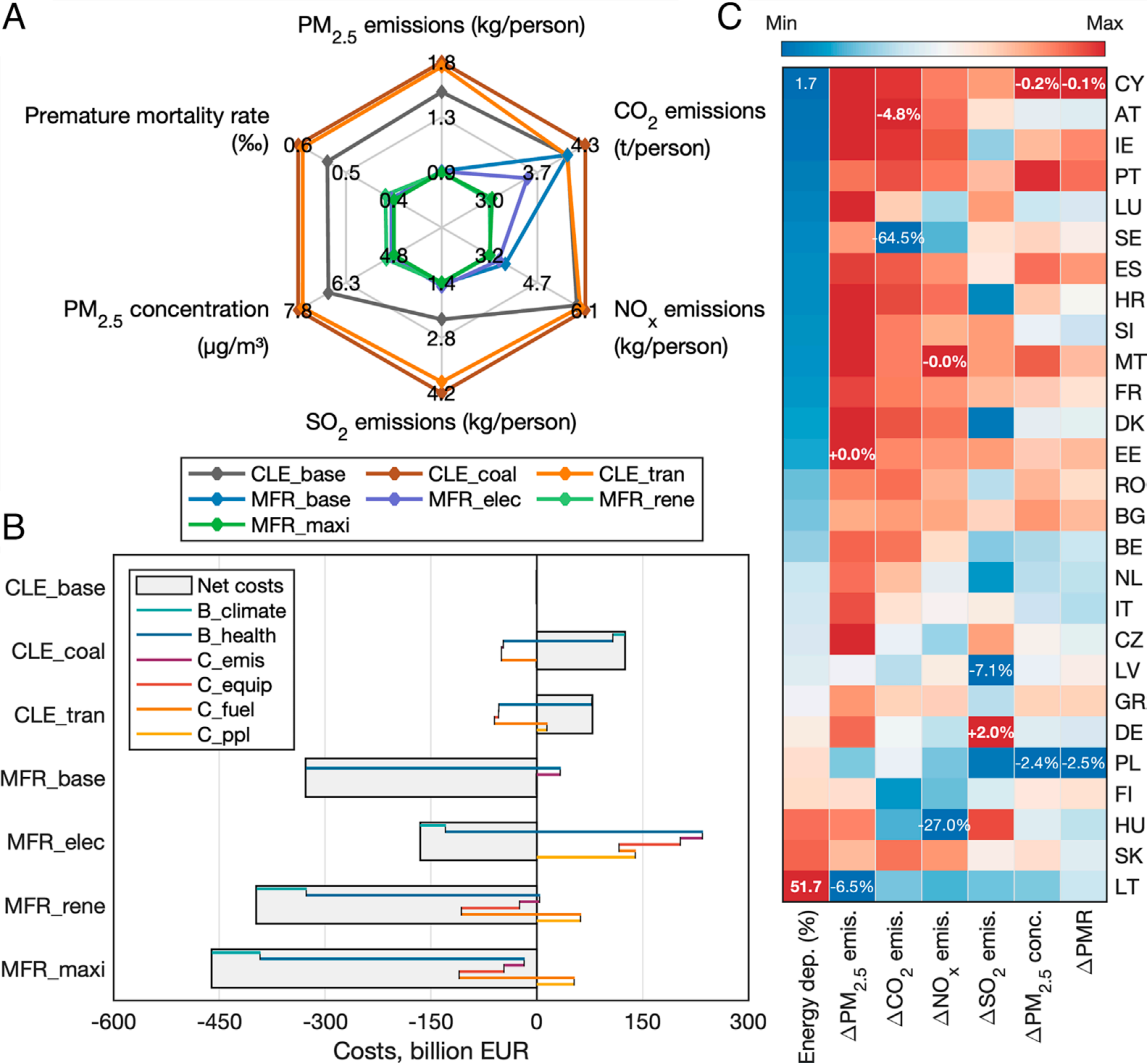
Costs and benefits of different scenarios across EU countries in 2050. (*A*) Effects of each scenario on air pollutant emissions (PM_2.5_, CO_2_, NO_x_, SO_2_), PM_2.5_ concentration, and premature mortality rate induced by PM_2.5_ and O_3_ exposure. Values present EU averages in 2050. (*B*) Net costs and disaggregated cost components across scenarios. Components include: C_ppl (power plant investment), C_fuel (fuel costs), C_equip (infrastructure costs), C_emis (emission control costs), B_health (monetized health benefits), and B_climate (monetized climate benefits). (*C*) Country-level energy dependency, and the changes from MFR_base to MFR_maxi in 2050 for air pollutant emissions (PM_2.5_, NO_x_, SO_2_), CO_2_ emissions, PM_2.5_ concentration, and premature mortality rate (PMR) in 2050. Countries are ordered by energy dependency. Color shading from blue (minimum) to red (maximum) represents the range of values within each row. Minimum and maximum values are marked with regular and bold fonts, respectively.

Fig. 3*A* shows the environment and health impacts for different scenarios in 2050. Compared to CLE_base, both CLE_coal and CLE_tran would increase the health burden in 2050 by 11,800 (8,900 to 14,700) cases and 10,000 (7,400 to 12,300) cases, respectively. Similarly, the saved fuel costs (49 (20 to 78) billion EUR and 75 (39 to 109) billion EUR) are not sufficient to offset those monetized excess premature mortality and led to additional costs in 2050 of 125 (90 to 179) billion EUR and 79 (40 to 117) billion EUR, respectively (Fig. 3*B*).

In the long term, stricter measures to mitigate air pollution-related health impacts also become feasible (31). The Maximum Technically Feasible Reduction (MFR) (31) scenario has been developed using the same energy demand as CLE but assuming a stringent policy requiring introduction of the best available technology for all economic activities generating emissions starting from 2020 (32), aligning with the EU’s clean air goals. We define this MFR scenario as MFR_base in our analysis. From MFR_base, the supply-side scenarios were developed by filling the energy gap with electricity (MFR_elec) or more renewables (MFR_rene) while the demand-side scenario was developed by reducing private transport demand with renewables filling the remaining energy gap (MFR_maxi). Note that, we did not combine coal-based strategies with MFR as the fossil fuel does not align with the clean air goals aimed by the MFR scenario. More details are described in *Materials and Methods* and *SI Appendix*, Supplementary Note 1.

Our results show that the substantial benefits would result from more ambitious renewable energy targets and less energy-consuming lifestyles in the future. Compared to CLE_base, MFR_elec, MFR_rene, and MFR_maxi would reduce premature mortality due to PM_2.5_ and O_3_ by 24,000 (16,000 to 31,000) cases, 22,000 (15,000 to 29,000) cases and 24,000 (17,000 to 32,000) in 2050, corresponding to monetized health costs of 364 (322 to 406) billion EUR, 331 (292 to 369) billion EUR and 374 (331 to 417) billion EUR, respectively (Fig. 3*B*). The strategies to close the energy gap could also reduce CO_2_ emission by 227 Mt, 440 Mt, and 426 Mt (equivalent to 0.5 t/person, 1.0 t/person, and 1.0 t/person, Fig. 3*A*), respectively, leading to monetized climate benefits of 37 (10 to 147) billion EUR, 71 (19 to 285) billion EUR, and 69 (18 to 276) billion EUR (Fig. 3*B*). Together with fuel cost savings, the health and climate benefits are sufficient to offset their additional costs for power generation in the three scenarios. As shown in Fig. 3*B*, compared to CLE_base, the total net benefits (including all six components) in 2050 are 165 (97 to 282) billion EUR for MFR_elec, 398 (295 to 608) billion EUR for MFR_rene, and 461 (360 to 663) billion EUR for MFR_maxi. Generally, from closing the energy gap, countries with higher initial energy dependency could gain more environmental and health benefits, more reduction of CO_2_ and air pollutant emissions and premature mortality rate (Fig. 3*C*). Eastern EU countries such as Poland, Latvia, Slovakia, and Hungary are more reliant on Russian imports and exhibit the largest benefits, highlighting their sensitivity to energy strategy choices (Fig. 3*C* and *SI Appendix*, Fig. S2).

### Accelerating the Energy Transition.

**Fig. 4.**
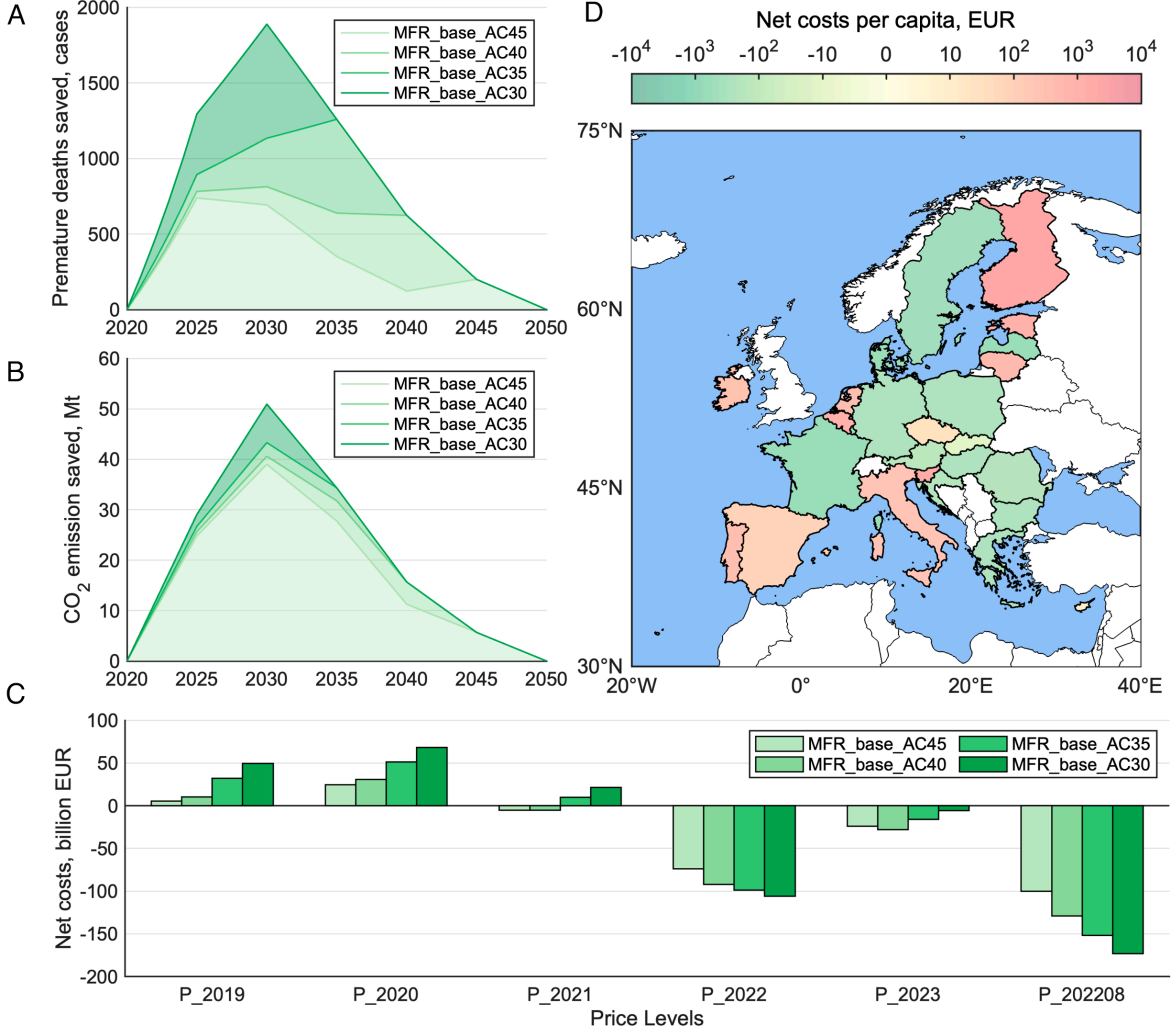
Net costs for accelerating the EU energy transition during the period of 2020 to 2050 under MFR pathway. (*A*) Reduced premature mortality attributable to air pollution (PM_2.5_ and O_3_) (*B*) Reduced CO_2_ emissions from 2020 to 2050 for the scenarios MFR_base_AC45, MFR_base_AC40, MFR_base_AC35, and MFR_base_AC30. (*C*) EU cumulative net costs from 2020 to 2050 for scenarios MFR_base_AC45, MFR_base_AC40, MFR_base_AC35, and MFR_base_AC30 at different price levels. (*D*) Country-level net costs per capita for scenarios MFR_base_AC40 at price level P_2023.

Incentivized by the rising energy import price, we further examine the possibility of accelerating energy transition in the EU based on its energy transition goals for 2050. Starting from the MFR_base scenario, we analyzed the effects of accelerating the energy transition by reaching 2050’s energy targets earlier by 5-y intervals: MFR_base_AC45, MFR_base_AC40, MFR_base_AC35, and MFR_base_AC30. For instance, in the MFR_base_AC40 scenario, we assume that the EU would reach the 2050 goals of electricity and energy structure in 2040, i.e., 10 y in advance. These early transition scenarios yield more cumulative health (Fig. 4*A*) and climate (Fig. 4*B*) benefits, but correspondingly increase electricity and renewable energy demands, thereby raising power generation and other associated costs. Evaluating whether these benefits outweigh the costs is crucial for identifying the optimal transition pathway. Thus, we employed the innovative integrated assessment framework combining WILIAM and GAINS to evaluate the net costs of the early transition. Moreover, we also considered the potential price volatility when analyzing the impacts of the rising energy prices on the net costs of early energy transition, by dividing six price levels from 2019 to 2023 in the fuel cost estimates (denoted as P_2019, P_2020, P_2021, P_2022, P_2023, and P_202208) (*SI Appendix*, Fig. S3).

Fig. 4*C* shows the net costs for accelerating the EU energy transition during the period of 2020 to 2050. At relatively low fuel price levels (P_2019, P_2020, and P_2021), almost all scenarios accelerating the energy transition by 5 to 20 y results in positive net costs (*SI Appendix*, Supplementary Note 3). This is because the additional investments in power plants and supporting infrastructure exceed the combined value of saved fuel costs and climate and health benefits. When fuel prices rise to P_2022 and P_2023, net costs drop to negative values (net benefits), indicating the combined values of saved fuel costs and climate and health benefits exceed the additional investments in power plants and infrastructure. Under P_2023, MFR_base_AC40 results in the lowest net cost, suggesting that initiating the transition either too early or too late may not be economically optimal. When energy prices spike to the P_202208 level, accelerating the energy transition yields even greater net benefits, and the lowest-cost scenario shifts to MFR_base_AC30. This suggests that once energy prices surpass a certain threshold, initiating the transition earlier becomes increasingly beneficial. Overall, our results show that rising energy prices not only enhance the cost-effectiveness of accelerating the energy transition in the EU but may also alter the optimal transition pathway.

The effects of accelerated energy transition exhibit large heterogeneity across countries. These country-level disparities indicate that the same transition pace can yield different outcomes depending on national circumstances. For example, at the price level P_2023, the EU experience an overall net benefit from accelerating the energy transition by 10 y (Fig. 4*C*), yet countries like Italy and Finland still face net costs (Fig. 4*D*) largely due to heavier burdens from power plant construction. These differences underscore the need to tailor transition strategies not only to collective EU goals but also to individual national contexts. Our country-level integrated assessment framework enables the evaluation of specific transition pathways for each country. The observed heterogeneity highlights the importance of high-resolution modeling in supporting more targeted, equitable, and effective policy design.

**Fig. 5.**
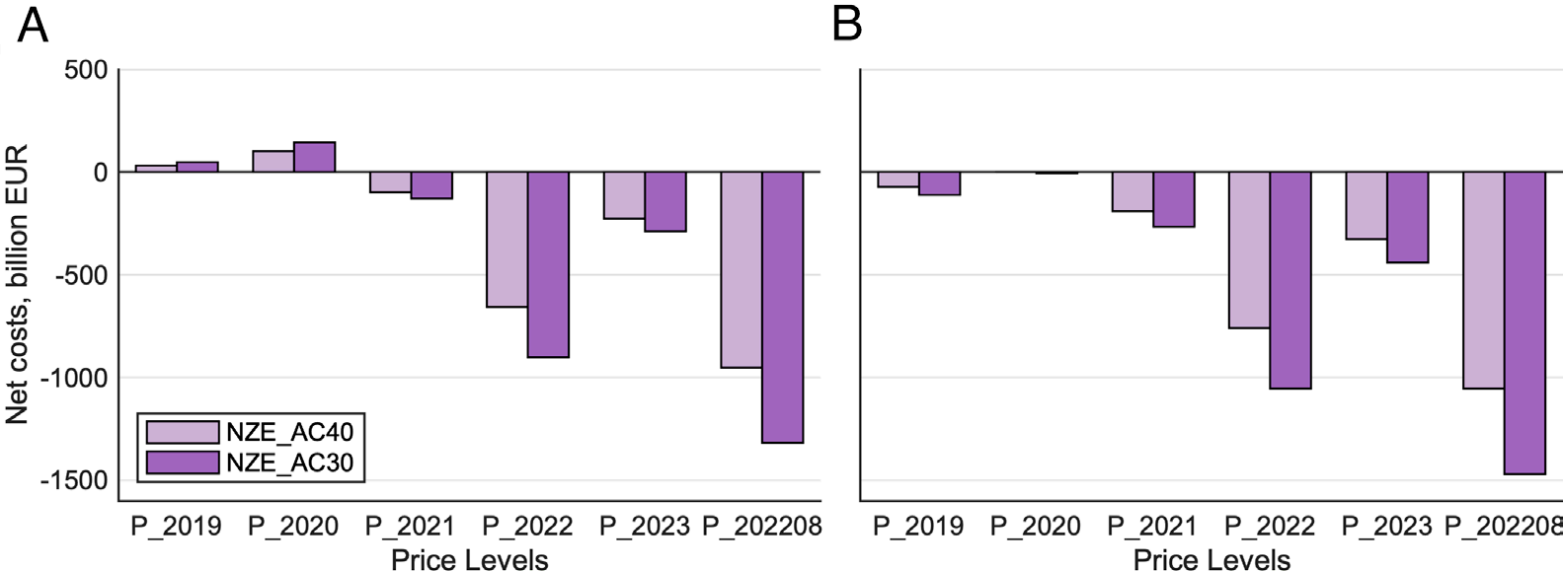
Net costs for accelerating the EU energy transition during the period of 2020 to 2050 under the NZE pathway. Accumulative net costs for NZE_AC40 and NZE_AC30 at six price levels by LCOE trends in LCOE_max (*A*) and LCOE_baseline (*B*). Six price levels are the annual average prices before the COVID-19 pandemic (P_2019), during the pandemic period (P_2020), in 2021 (P_2021), in 2022 (P_2022), in 2023 (current average prices) (P_2023) and the high price stage in August 2022 (P_202208).

Besides MFR scenarios, we also evaluated the effects of energy transition acceleration under the Net Zero Emission (NZE) scenario, which has more ambitious climate targets and requires a substantial renewable energy expansion (33, 34). In this case, the renewable energy share in 2050 increases dramatically to about 80% (33). Fig. 5*A* shows the net costs for accelerating the EU energy transition under the NZE scenario. We analyzed NZE_AC40 and NZE_AC30 for reaching the energy targets in 2050 by 10 y and 20 y earlier, respectively. Similarly, at relatively low fuel price levels such as P_2019 and P_2020, accelerating energy transition incurs net costs, while at relatively high price levels, these net costs shift into substantial net benefits. While MFR_base_AC30 only becomes the most beneficial scenario at the extreme high fuel price level (P_202208), NZE_AC30 starts to outperform NZE_AC40 already from the moderate fuel price level (P_2021). The ~80% high renewables share in the NZE pathway largely explains the greater net benefits of accelerating the energy transition under rising energy prices, making the early transition for 20 y more beneficial than the 10 y. Again, this suggests that rising energy prices can significantly enhance the cost-effectiveness of accelerating the energy transition and alter the optimal transition pathway, even under the NZE scenario.

Under NZE scenarios, EU countries would face a sudden expansion of renewables when accelerating the energy transition, potentially increasing LCOE values due to increased curtailments. Capturing these changes is essential but challenging and has been often overlooked in previous studies. The WILIAM model explicitly incorporates flexibility management strategies, curtailment, and related factors, enabling a more robust evaluation of the rapid renewable energy expansion (29). When dynamically considering these effects, the LCOE for nuclear and renewables under an intensive expansion may change. For example, the LCOE for wind and solar PV would rise up to 20% (*SI Appendix*, Fig. S4). The dynamic LCOEs have been taken into account in our aforementioned calculations. Fig. 5 compares net cost estimates with (Fig. 5*A*) and without (Fig. 5*B*) accounting for curtailment and related effects due to rapid renewables expansion. Neglecting these effects leads to an underestimation of net costs by roughly 100 billion EUR for NZE_AC40 and 150 billion EUR for NZE_AC30. This would lead to overly optimistic estimation that accelerating the NZE transition is cost-effective under almost all price levels (Fig. 5*B*). In contrast, incorporating these effects (Fig. 5*A*) enables a more realistic assessment of its economic implications.

## Discussion

The EU’s previous high reliance on imported energy has led to a significant energy supply gap. In this study, we assessed potential strategies to alleviate the energy crisis by quantifying their costs and environmental-health-climate impacts using an innovative assessment framework integrating GAINS and the system dynamic WILIAM models. Some countries in the EU have made efforts since early 2022 to reduce their energy demands and find alternative solutions for natural gas energy sources. For example, German governments passed amendments to the Energy Industry Act such as the Act on the Maintenance of Substitute Power Stations (35) to replace natural gas consumption by other energy sources including clean energy and fossil fuels in the short term in reaction to the current gas supply situation (36). Across the EU, a total of 26 coal plants with a combined generation capacity of 11GW were reactivated and placed on emergency standby in 2022 (37). While these measures can partially offset short-term energy supply fluctuations, we show that they increase either economic or public health burdens, underscoring the need for more fundamental and structural transitions in the future.

Among long-term strategies, our results show that promoting electrification and renewables with stricter pollution controls in the long-term yields substantial health benefits, more than offsetting the additional costs. Our sensitivity analysis confirms that this conclusion holds across all price levels: CLEs consistently result in net costs, while MFRs yield net benefits (*SI Appendix*, Fig. S5). Therefore, it would be beneficial for the EU countries to develop or update their long-term energy strategies without delay. However, these results should also be interpreted in their whole macroeconomic dimension. By definition, GDP can be measured as all the expenditures made by economic agents in an economy. Thus, net cost savings alone could lead, in the short term, to GDP losses if not offset by productivity gains and imports substitution by domestic production. Consequently, it is important to discuss what to do with the generated savings. First, it could finance industrial policy oriented at reshoring of critical supply chains for green technologies in the EU. Second, it could finance demand-sided policies such as mass public transport that would also further expand health benefits. Finally, it could provide the resources to fund the energy transition in the Global South, as it is already committed by the EU.

Moreover, rising energy prices can incentivize the acceleration of the EU’s energy transition and alter the optimal transition pathway. Our results demonstrate that despite increased infrastructure costs, an early achievement of the 2050 energy structure goals can lead to net benefits. These benefits would be further amplified by the increasing fuel prices, indicating that proactive measures modulating fossil fuel prices, such as adjusting carbon taxes, can economically incentivize the transition to clean energy. By dynamically accounting for curtailment, transmission and storage constraints, spatiotemporal resource heterogeneity, and techno-economic feedbacks, particularly on LCOE, we find that the rising energy prices may make the early energy transition cost-effective even in the more ambitious decarbonization pathway (NZE) (33). Ignoring the fluctuation of LCOE would lead to an underestimate of the costs associated with renewable energy expansion. Future work incorporating material constraints, biophysical potential, and grid-level dynamics, including hourly dispatch and spatial balancing could further improve the renewable energy cost and deployment estimates especially in the analysis of net-zero strategies (22, 38).

More broadly, our study demonstrates that, when energy systems are exposed to shocks such as supply constraints, price volatility, or crisis conditions, cost–benefit assessments of energy-transition strategies can favor systemic changes in strategy portfolios and transition pathways, with implications that extend well beyond marginal adjustments to existing strategies. More ambitious renewable energy targets are expected to stimulate technological innovation in the energy sector, which may further boost productivity and economic growth, creating new opportunities in the labor market and driving broader economic development. In fact, a key measure in the REPowerEU plan is to increase renewable energy capacities (9), which can serve as a crucial starting point for accelerating the EU’s energy transition. In addition, energy diversity and independence are critical for ensuring energy security (39). The EU’s reliance on energy imports has exacerbated the current crisis, highlighting the need for countries with high energy dependency to consider price volatility and supply stability in long-term policymaking. Tariff increases on key energy technologies and components may further complicate this process by raising the costs of renewable energy deployment, potentially slowing down the pace of the energy transition and increasing the overall transition costs. Additionally, promoting diversity in energy import sources and advancing electrification can lead to a more resilient energy system, and an early transition toward cleaner energy can be justified in terms of cost-effectiveness. By diversifying energy markets and accelerating energy transition, EU countries can make significant progress toward a more energy-secure and cleaner future. Our results also offer valuable insights for regions like Africa and South Asia, where growing energy demands and air pollution highlight the need for comprehensive cost assessments to guide sustainable energy transitions.

## Materials and Methods

### Scenario Description.

To evaluate the impacts of different energy transition measures in the EU, we developed a set of counterfactual scenarios as a policy stress test under abrupt energy-supply disruption. This framework is designed to compare the implications of alternative short-term coping measures and long-term transition pathways under transparent assumptions, rather than to reproduce full short-run market equilibrium adjustments. Specifically, we consider a number of scenarios representing or adjusted based on the current legislation (CLE), maximum technically feasible reduction (MFR) and net-zero emission (NZE) pathways (Table 1). CLE denotes the current legislation scenario developed by the Evaluating the Climate and Air Quality Impacts of Short-Lived Pollutants project (ECLIPSE V6b) (24, 40), based on the New Policies scenario in International Energy Agency (IEA) World Energy Outlook (WEO) 2018 (41), aligning broadly with the IEA’s mid-range outlook (31). This scenario assumes the successful implementation of existing air pollution legislation and incorporates anticipated effects from announced energy, climate, and air quality policies, reflecting established targets and strategic plans (25). We define this CLE scenario as CLE_base in our analysis, serving as the baseline projection of energy and emissions under current policies without additional interventions or policy expansions. We use Eurostat data (2) to quantify each country’s energy gap by excluding Russian imports and evaluate measures from supply and demand sides to close the gap. The supply-side scenarios are CLE_coal and CLE_biom, representing scenarios where coal or biomass is used to fill the energy gap, respectively; and demand-side scenarios are CLE_heat and CLE_tran, which represents lowering indoor heating temperatures by 3â and reducing private transportation demand by 20%, respectively. Because heating degree days (HDDs) correlate with heating demand (42), we use HDDs as a proxy for reduced heating needs. HDDs are calculated from daily temperatures in the latest European Centre for Medium-range Weather Forecasts (ECMWF) climate reanalysis (43). When a scenario cannot fully meet the energy gap with the chosen measure, coal is used as a substitution fuel. The detailed construction of the scenarios is in *SI Appendix*, Supplementary Note 1.

**Table 1.** Scenario description

Scenario	Description	S-Term (2025)	L-Term (2050)	AC-Transition
CLE_base	Current Legislation^*^,^†^	✓	✓	
CLE_coal	Filling the energy gap with hard coal	✓	✓	
CLE_biom	Filling the energy gap with biofuels	✓		
CLE_heat	Lowing indoor heating temperature by 3 °C and filling the gap using hard coal	✓		
CLE_tran	Reducing private transportation demand by 20% and filling the gap using hard coal	✓	✓	
MFR_base	Maximum Feasible Reduction^*^,^‡^		✓	✓
MFR_elec	Electrification with the original electricity generation mix		✓	
MFR_rene	Electrification using renewable energy for the additional electricity demand		✓	
MFR_maxi	Maximum actions, MFR_rene with the 20% reduction in private transportation demand		✓	
MFR_base_ACyy	Achieving 2050 MFR_base energy mix by 20yy (2030, 2035, 2040, 2045)			✓
NZE	Net Zero Emissions^§^			✓
NZE_ACyy	Achieving 2050 NZE energy mix by 20yy (2030, 2040)			✓

Three scenario groups are examined: CLE (current legislation), MFR (maximum feasible reduction), and accelerated energy transition based on MFR_base and NZE pathways. The rightmost columns indicate which scenarios are analyzed in each time period: short-term (S-Term), long-term (L-Term), and accelerated transition (AC-Transition).

^†^The CLE scenario assumes the existing air quality legislation is fully implemented and enforced.

^‡^The MFR assumes that all technologically feasible emissions reduction measures are implemented.

^§^data source: Boitier et al. (33).

^*^These scenarios were developed in the framework of the ECLIPSE (Evaluating the Climate and Air Quality Impacts of Short-Lived Pollutants) project (IIASA, 2019). They include both climate and regional air quality policies for the emissions of air pollutants.

For the MFR scenarios, MFR_base is also developed by the project ECLIPSE (24, 40). It follows the same energy pathway as CLE_base but incorporates stricter emission controls with higher emission reduction technology penetration [for details see “control_strategy” in the data repository and (44)]. Similarly, we also develop supply- and demand-side scenarios to close the energy gap. The supply-side scenarios—MFR_elec and MFR_rene—describe two pathways for closing the energy gap via electrification: The former using current energy mix, and the latter relying solely on renewable sources. The demand-side scenario is MFR_maxi, representing a 20% reduction in private transportation demand with renewables filling the remaining energy gap. In this case, a 20% reduction in private transportation demand alone cannot close the energy gap; therefore, the so-called demand-side measure is also paired with additional renewable energy supply. Lowering thermostat conflicts with Sustainable Development Goals (SDGs) and sustained exposure to cold indoor temperatures is associated with increased health risks (45–47). Thus, lowering the thermostat is not treated as a sustainable measure, and is excluded from the long-term strategies. All scenarios ensure internal consistency by closing the energy gap either through electrification with defined energy mixes or through demand reduction supplemented by renewable electricity. From MFR_base, we also develop a set of scenarios with different paces of accelerating the energy transition: MFR_base_AC45, MFR_base_AC40, MFR_base_AC35, and MFR_base_AC30, which represents achieving the 2050 energy targets by 2045, 2040, 2035, and 2030, respectively.

The NZE scenario following a carbon neutrality pathway is taken from the work of Boitier et al. (33). They developed a NZE Benchmark medium scenario, which outlines a balanced pathway for the EU to achieve net-zero emissions by 2050, emphasizing gradual decarbonization across sectors, increased renewable energy adoption, and moderate reliance on carbon capture technologies while maintaining economic stability (33). We take the NEMESIS model results of the NZE Benchmark medium scenario as our NZE scenario. Similarly, we develop NZE_AC40 and NZE_AC30 for achieving the 2050 energy targets by 2040 and 2030, representing accelerating the energy transition by 10 y and 20 y, respectively.

### WILIAM Model.

The WILIAM (“Within limits”) Integrated Assessment Model is a system dynamics policy-simulation model based on the MEDEAS model (48), which considers the dynamic relationships among demography, society, economy, energy, materials, land and water, etc. (29). A detailed description of the model is available in project reports (49, 50) and in the model’s wiki (29). Particularly, data sources for each module within WILIAM are listed on the respective wiki pages (29). *SI Appendix*, Supplementary Note 4 is a brief guide to the model. Recent publications have employed the WILIAM model to analyze energy transition from a multisectoral and multiregional perspective (51, 52). Here, we apply WILIAM v1.3 (29) to estimate LCOE trends for renewable energy and nuclear energy in the EU from 2020 to 2050. For renewable energy, we have considered the following categories: wind onshore, solar open space PV, hydropower dammed, and geothermal energy.

It is important to consider LCOE variation because aggressively promoting renewables—particularly on a carbon-neutral pathway—can raise their levelized costs, yet most IAMs overlook how these potential cost variabilities are affected by the energy system (30, 53). In WILIAM, renewable LCOE is calculated within the Energy module, with dynamic links to other modules such as economy, land use, and materials [for details see WILIAM’s wiki (29)]. Curtailment is a key driver of LCOE change under rapid renewable expansion, and is parametrically estimated as a dynamic reduction in full-load hours based on renewable penetration, system flexibility, storage deployment, and related factors (*SI Appendix*, Supplementary Note 5). WILIAM emulates the hourly resolution EnergyPLAN model to capture the relationship between curtailment and influencing factors, particularly capacity expansion, allowing the model to account for structural limits to flexibility such as insufficient storage or delayed grid upgrades (54). The reduced full-load hours are directly reflected in LCOE calculations, alongside other factors including spatiotemporal resource heterogeneity, climate-related impacts and CO_2_ prices, enabling WILIAM to capture key system-driven cost dynamics for renewable LCOE.

*SI Appendix*, Fig. S4 compares the simulated LCOE trends with and without a rapid renewable expansion for wind and solar PV. As shown in Fig. 1, the trends of LCOE for various renewable energy sources from WILIAM are considered in the cost assessments for power plants expansion.

### GAINS Model.

The GAINS model for Europe (v4.02a) is developed by International Institute for Applied Systems Analysis (IIASA) (40). It is a comprehensive energy-emission-impacts assessment model including activities, emissions, air quality, health, and climate impacts, as well as emission control measures and costs (55). It provides pathways from policy measures to air pollution, public health, and climate impacts by analyzing data associated with economic and agricultural development, emission control and cost information, and so on (56). It has been widely used to address future pathways and air pollution mitigations and its impacts on human health and climate in Europe (55–57) and worldwide (58–60). The GAINS model also contains the pathways such as Current LEgislation (CLE) strategy and Maximum Feasible Reduction (MFR) strategy from the ECLIPSE project (24). The GAINS simulations were implemented using sector-specific configuration settings and input files, as detailed in *SI Appendix*, Table S1.

The GAINS’s emissions are developed by a bottom–up method for eight major air pollutants including ammonia (NH_3_), carbon dioxide (CO_2_), methane (CH_4_), nitrogen oxides (NO_x_), particulate matter (TSP, PM_10_, PM_2.5_, and PM_1_), sulfur dioxide (SO_2_), volatile organic compounds (VOC), and carbon monoxide (CO), across six major activity sectors including power generation, industry, agriculture, transportation, waste, and domestic sectors. Ambient concentrations are then simulated at 28 km x 28 km resolution in the GAINS model via a reduced-form method (61) based on results of the European Monitoring and Evaluation Programme (EMEP) chemistry transport model (62).

We assessed health impacts as premature mortality due to ambient PM_2.5_ and ground-level ozone exposure. PM_2.5_ concentration is directly from GAINS. PM_2.5_ related premature mortality was estimated using the Meta-Regression-Bayesian, Regularized, Trimmed splines (MRBRT) risk model (63) from the Global Burden of Disease (GBD) 2019 (64). Ozone-related mortality was calculated in GAINS based on the APHENA study (65).

### Monetization of Costs and Benefits.

We considered power generation costs, fuel costs, infrastructure costs, emission control costs, social costs of carbon and the monetized health impacts to estimate the net costs for each scenario. We calculated power generation costs by multiplying the LCOE by the additional electricity demand for each scenario. For renewable and nuclear energy, LCOE in 2020 were drawn from IEA at a 7% interest rate, and WILIAM was used to calculate their trends till 2050. For other energy types, LCOE values in 2020, 2030, and 2050 were drawn from IEA at a 7% interest rate (31). The LCOE reflects the discounted lifetime cost of a power plant—including capital expenditures, fixed and variable operations and maintenance, fuel, financing, and decommissioning—per unit of electricity generated.

We calculated fuel costs by multiplying fuel prices of biomass and fossil fuels by the corresponding changes in final energy demand with respect to the baseline. For fuel prices, we combined the import price index in Germany (6) with the IEA’s fuel price data for the EU (31), and country-level prices for coal, wood, oil, and natural gas gathered from government reports, websites, and trade data. To evaluate the impacts of rising energy prices, we defined six price levels corresponding to the annual average prices before the pandemic (P_2019), during the pandemic period (P_2020), in 2021 (P_2021), in 2022 (when the effects of the geopolitical situation were most severe) (P_2022), in 2023 (P_2023) and the high price stage in August 2022 (P_202208). We included August 2022 because it marks the peak of geopolitically driven energy prices. In that month, the import prices surged to 5.1 times for coal, 3.2 times for diesel and oil, and 9.5 times for natural gas compared to 2020 levels.

For infrastructure costs, we considered electric grid infrastructure (66), industrial electric boilers (67), residential electric devices and upgrading (68), and charging devices for electric vehicles (69). These infrastructure costs are essential complements to power generation costs because expanding electrification requires upgrading and expanding networks, end-use equipment, and charging systems to deliver and utilize additional electricity reliably and efficiently.

Emission control costs for air pollutants were calculated by GAINS, incorporating country-specific adjustments for local labor, by-product revenues, and other parameters (70). Because emission control measures are typically deployed with a profit motive, we apply a 10% interest rate.

We monetized health impacts by multiplying premature mortality by country-specific values of a statistical life (VSL) (71). We adopt a VSL of 5.3 million EUR for Germany at a 5% interest rate (71) and derive the VSLs for other EU countries via the benefit transfer approach (72), applying a personal income elasticity of 0.5. VSLs are scaled to GDP per capita from the shared socioeconomic pathway SSP2 projections (73) and converted to EUR in 2021 with International Monetary Fund (IMF) inflation rates (74).

To monetize climate impacts, we applied the social cost of carbon dioxide (SC-CO_2_), which quantifies the societal damages caused by unit incremental CO_2_ emissions (75). The SC-CO_2_ ranges from $44 to $413 per tCO_2_, with the recommended value of $185 per tCO_2_ (75). The detailed methods are in *SI Appendix*, Supplementary Note 5. The results are presented in EUR in 2021.

### Uncertainty Analysis.

A Monte Carlo simulation (1,000 iterations) was conducted to quantify uncertainties in relative risks for health impacts, the value of a statistical life (VSL), the social cost of carbon dioxide (SC-CO_2_), fuel prices, and the LCOE, expressed as 95% CI (CIs). Relative risk uncertainties were derived from MR-BRT estimates in GBD 2019 (63); VSL was varied by ±50%; and SC-CO_2_ uncertainties, represented by the 5 to 95% quantile range, were taken from Rennert et al. (75). Fuel price uncertainty was based on observed monthly fluctuations; LCOE uncertainty was based on aggregated data from multiple sources, listed in the data repository with sources.

### Sensitivity Analysis.

We performed a sensitivity analysis on six key input parameters, including LCOE, discount rate for equipment costs, elasticity factor, discount rate for SC-CO_2_ and VSL. For the LCOE we tested ±50% around the baseline (i.e. 50%, 100% and 150% of the central estimate); for the discount rate we compared 4%, 7% and 10%; for the elasticity factor in VSL, we used 0.3, 0.5, and 1.0; for the SC-CO_2_ we applied values at discount rates of 1.5%, 2% and 3% (75); for emission control costs we applied the results from the GAINS model at the interest rate 4%, 10% and 20% (40); and for the VSL we considered 50%, 100% and 150% of our reference value. The detailed results are in *SI Appendix*, Supplementary Note 6. We further performed a robustness analysis with an augmented WILIAM framework to examine endogenous fuel price responses to changes in energy consumption and supply strategies. The resulting price fluctuations had only negligible effects on net costs and did not alter our conclusions (*SI Appendix*, Supplementary Note 7).

## Supplementary Material

Appendix 01 (PDF)

## Data Availability

Model, parameters and results data have been deposited in Zenodo (76). Study data are included in the article and/or *SI Appendix*.
