# Supplementary material for: Rethinking energy transition strategies for the European Union amid rising energy prices
Source: Proc Natl Acad Sci U S A. 2026 Jun 1;123(23):e2609606123. doi: 10.1073/pnas.2609606123 (PMC13250444; doi:10.1073/pnas.2609606123)
Supplement: Supplementary file 1 — Appendix 01 (PDF) [file pnas.2609606123.sapp.pdf]

PNAS

Supporting Information for

Rethinking Energy Transition Strategies for the European Union amid Rising Energy Prices

Wenjun Meng[1,2], Jaime Nieto[3], Dabo Guan[4], Jing Meng[5], Robert Sander[6], Ulrich Pöschl[7], Klaus Hubacek[8], Hang Su[9*], Shu Tao[2,10], Yafang Cheng[1*]

[1] Aerosol Chemistry Department, Max Planck Institute for Chemistry, 55128 Mainz, Germany
[2] College of Urban and Environmental Sciences, Peking University, 100871 Beijing, China
[3] Research Group on Energy, Economy and System Dynamics, Escuela de Ingenierías Industriales, University of Valladolid, Paseo del Cauce s/n, 47011 Valladolid, Spain
[4] Department of Earth System Science, Tsinghua University, 100084 Beijing, China
[5] The Bartlett School of Sustainable Construction, University College London, London WC1E 7HB, United Kingdom
[6] International Institute for Applied Systems Analysis (IIASA), Schlossplatz 1, A-2361 Laxenburg, Austria
[7] Multiphase Chemistry Department, Max Planck Institute for Chemistry, 55128 Mainz, Germany
[8] Integrated Research on Energy, Environment and Society (IREES), Energy and Sustainability Research Institute Groningen, University of Groningen, Groningen, Netherlands
[9] Institute of Atmospheric Physics, Chinese Academy of Sciences, 100029 Beijing, China
[10] School of Environmental Science and Engineering, Southern University of Science and Technology, 518055 Shenzhen, China

* Corresponding author: Yafang Cheng and Hang Su.

Email: yafang.cheng@mpic.de, suhang@mail.iap.ac.cn

This PDF file includes:

Supplementary Note 1 to 7
Figures S1 to S16
Table S1 to S2
SI References

Supplementary Note 1

Scenario setup

Here, each scenario contains the information of country-level energy consumption, denoted as $E^{scenario}$, for all sectors and energy types. Taking the energy consumption $E^{scenario}$ as input, the corresponding costs and benefits can be calculated by the comprehensive framework shown in Fig. 1. In the following, we describe how to calculate the country-level energy consumption $E^{scenario}$ for each scenario. Note that $E^{scenario}(t, c, s, f)$ is a function of year t, EU country c, sector s (e.g., industry, transportation, residential, commercial, services and agriculture sectors) and energy type f (e.g., coal, oil, gas, biomass or electricity).

1.1 Energy consumption for scenarios closing the energy gap

As explained in the main text, we evaluated the costs and benefits of different scenarios to close the energy gap. These scenarios are developed by first quantifying each country's energy gap from the baseline scenario and then applying alternative measures on both the supply and demand sides.

Baseline scenarios

The current legislation (CLE) and maximum feasible technical reduction (MFR) scenarios are taken as baseline scenarios here. They were developed in project ECLIPSE (Evaluating the Climate and Air Quality Impacts of Short-Lived Pollutants) V6b by IIASA (for details see descriptions in IIASA (1)).

Accordingly, $E^{\text{CLE\_base}}(t, c, s, f)$ and $E^{\text{MFR\_base}}(t, c, s, f)$ represent the energy consumption of the baseline scenarios CLE_base and MFR_base, respectively. The data of $E^{\text{CLE\_base}}$ and $E^{\text{MFR\_base}}$ are taken from the well-established scenarios current legislation CLE (ECLIPSE_V6b_CLE) and maximum feasible technical reduction MFR (ECLIPSE_V6b_MFR), which can be downloaded directly from the GAINS model (2). Years $t = 2025$ and $t = 2050$ are used for the short- and long-term scenarios, respectively (Table 1).

Energy gaps

The energy gap $E_{\text{gap}}(t, c)$ is determined by the Russian energy imports:

$$E_{\text{gap}}(t, c) = \sum_{f \in \mathcal{F}_{\text{fossil}}} E_{\text{gap}}(t, c, f) \quad (1)$$

$$E_{\text{gap}}(t, c, f) = E_{\text{RussiaImport}}(t, c, f),\ f \in \mathcal{F}_{\text{fossil}} \quad (2)$$

where $\mathcal{F}_{\text{fossil}} = \{coal, oil, gas\}$ is the set of fossil fuels.

For easy understanding of the following calculations, we also define the intermediate parameters $E_{\text{remain}}^{\text{CLE\_base}}$ and $E_{\text{remain}}^{\text{MFR\_base}}$. They represent the remaining energy consumption

after stopping the Russian energy imports. Note that the gap is allocated to each sector using its current share in the total energy consumption.

$$E_{\text{remain}}^{\text{CLE\_base}}(t,c,s,f) = E^{\text{CLE\_base}}(t,c,s,f) - E_{\text{gap}}(t,c,f) \times g_s\left(E^{\text{CLE\_base}}(t,c,s,f)\right) \quad (3)$$

$$E_{\text{remain}}^{\text{MFR\_base}}(t,c,s,f) = E^{\text{MFR\_base}}(t,c,s,f) - E_{\text{gap}}(t,c,f) \times g_s\left(E^{\text{MFR\_base}}(t,c,s,f)\right) \quad (4)$$

here $g_s\big(E(s)\big)$ is a function to calculate the fraction of energy consumption $E(s)$ of one specific sector in the sum of energy consumption of all sectors.

$$g_s\big(E(s)\big) = \frac{E(s)}{\sum_s E(s)} \quad (5)$$

Supply-side scenarios

In CLE-derived scenarios, we explored closing the energy gap with coal (CLE_coal) or biomass (CLE_biom).

For the **CLE_coal scenario**, the energy consumption $E^{\text{CLE\_coal}}(t,c,s,f)$ is calculated by filling the energy gap with coal:

$$E^{\text{CLE\_coal}}(t,c,s,f) = E_{\text{remain}}^{\text{CLE\_base}}(t,c,s,f),\ f \neq \text{coal} \quad (6)$$

$$E^{\text{CLE\_coal}}(t,c,\text{s},\text{coal}) = E_{\text{gap}}(t,c) \times g_s\left(E^{\text{CLE\_base}}(t,c,\text{s},\text{coal})\right) + E_{\text{remain}}^{\text{CLE\_base}}(t,c,s,\text{coal}) \quad (7)$$

For the **CLE_biom scenario**, the energy consumption $E^{\text{CLE\_biom}}(t,c,s,f)$ is calculated by filling the energy gap with biomass:

$$E^{\text{CLE\_biom}}(t,c,s,f) = E_{\text{remain}}^{\text{CLE\_base}}(t,c,s,f),\ f \neq \text{biom} \quad (8)$$

$$E^{\text{CLE\_biom}}(t,c,\text{s},\text{biom}) = E_{\text{gap}}(t,c) \times g_s\left(E^{\text{CLE\_base}}(t,c,s,\text{biom})\right) + E_{\text{remain}}^{\text{CLE\_base}}(t,c,s,\text{biom}) \quad (9)$$

In MFR-derived scenarios, we explored closing the energy gap via electrification: with current energy mix (MFR_elec), or with renewable sources (MFR_rene).

For the **MFR_elec scenario**, the energy consumption $E^{\text{MFR\_elec}}(t,c,s,f)$ is calculated by filling the energy gap with electricity.

$$E^{\text{MFR\_elec}}(t,c,s,f) = E_{\text{remain}}^{\text{MFR\_base}}(t,c,s,f),\ f \neq \text{elec} \quad (10)$$

$$E^{\text{MFR\_elec}}(t,c,\text{s},\text{elec}) = E_{\text{gap}}(t,c) \times g_s\left(E^{\text{MFR\_base}}(t,c,s,\text{elec})\right) + E_{\text{remain}}^{\text{MFR\_base}}(t,c,s,\text{elec}) \quad (11)$$

Unlike other energy types, the costs of electricity depend on how it is generated, e.g., by renewable energy, coal or gas etc. Thus, using electricity to fill the gap requires additional information about how it is generated. In the scenario MFR_elec, we assume that electricity is generated by the same energy mix as in MFR_base.

$$E_{\text{eg}}^{\text{MFR\_elec}}(t,c,f_{\text{eg}}) = E_{\text{gap}}(t,c) \times g_{f_{\text{eg}}}\left(E_{\text{eg}}^{\text{MFR\_base}}(t,c,f_{\text{eg}})\right) + E_{\text{eg}}^{\text{MFR\_base}}(t,c,f_{\text{eg}}) \quad (12)$$

where $E_{\text{eg}}(t,c,f_{\text{eg}})$ is the electricity generated for the year t, country c and electricity-generating energy type $f_{\text{eg}} \in \mathcal{F}_{\text{eg}} = \{\text{rene}, \text{nucl}, \text{coal}, \text{oil}, \text{gas}, \text{biom}\}$. Note that E_{eg} denotes the amount of electricity output from a certain energy source, not the amount of energy consumed. $g_{f_{\text{eg}}}$ calculates the fraction of electricity generation $E(f_{\text{eg}})$ of one specific electricity-generating energy type in the sum of electricity generation of all sectors.

$$g_{f_{\text{eg}}}\left(E(f_{\text{eg}})\right) = \frac{E(f_{\text{eg}})}{\sum E(f_{\text{eg}})} \quad (13)$$

Assuming the balance of electricity generation and consumption, we have

$$\sum_{s} E(t,c,s,elec) = \sum_{f_{\text{eg}}} E_{\text{eg}}(t,c,f_{\text{eg}}) \quad (14)$$

For the **MFR_rene scenario**, we assume that the energy gap is filled with electricity generated by renewable energy. We then have,

$$E^{\text{MFR\_rene}}(t,c,s,f) = E_{\text{remain}}^{\text{MFR\_base}}(t,c,s,f),\ f \neq \text{elec} \quad (15)$$

$$E^{\text{MFR\_rene}}(t,c,\text{s},\text{elec}) = E_{\text{gap}}(t,c) \times g_{s}\left(E^{\text{MFR\_base}}(t,c,s,\text{elec})\right) + E_{\text{remain}}^{\text{MFR\_base}}(t,c,s,\text{elec}) \quad (16)$$

$$E_{\text{eg}}^{\text{MFR\_rene}}(t,c,\text{rene}) = E_{\text{gap}}(t,c) + E_{\text{eg}}^{\text{MFR\_base}}(t,c,\text{rene}) \quad (17)$$

Demand-side scenarios

In CLE-derived scenarios, we explored reducing the energy gap by lowering indoor heating temperatures by 3℃ (CLE_heat) and reducing transportation demand by 20% (CLE_tran), respectively.

For the **CLE_heat scenario**, the energy consumption $E^{\text{CLE\_heat}}(t,c,s,f)$ is calculated by reducing the energy gap through a 3 ℃ decrease in indoor heating temperatures and filling the remaining energy gap with coal.

The heating demand reduction $\Delta E^{\text{CLE\_heat}}(t,c)$ is calculated by lowering heating by 3℃ for each heating day.

$$\Delta E^{\text{CLE\_heat}}(t,c) = \frac{3HD(c)}{HDD(c)} \sum_{f} E^{\text{CLE\_base}}(t,c,\text{heat},f) \quad (18)$$

where $HD(c)$ represents the number of days that require heating for country c, and $HDD(c)$ represents the heating degree days for country c (for details see Eurostat (3)).

When the reduced demand cannot fully close the energy gap, the remaining energy gap is:

$$E_{\text{gap}}^{\text{CLE\_heat}}(t,c) = E_{\text{gap}}(t,c) - \Delta E^{\text{CLE\_heat}}(t,c) \tag{19}$$

Using coal as a substitution fuel and adopting the same approach as in Equations (6) and (7), we can calculate $E^{\text{CLE\_heat}}$:

$$E^{\text{CLE\_heat}}(t,c,s,f) = E_{\text{remain}}^{\text{CLE\_base}}(t,c,s,f),\ f \neq \text{coal} \tag{20}$$

$$E^{\text{CLE\_heat}}(t,c,\text{s},\text{coal}) = E_{\text{gap}}^{\text{CLE\_heat}}(t,c) \times g_s\left(E^{\text{CLE\_base}}(t,c,\text{s},\text{coal})\right) + E_{\text{remain}}^{\text{CLE\_base}}(t,c,s,\text{coal}) \tag{21}$$

For the **CLE_tran scenario,** the energy consumption $E^{\text{CLE\_tran}}(t,c,s,f)$ is calculated by reducing the energy gap through a 20% reduction in transportation demand and filling the remaining energy gap with coal. The remaining energy gap is,

$$E_{\text{gap}}^{\text{CLE\_tran}}(t,c,f) = E_{\text{gap}}(t,c,f) - 0.2 \cdot E^{\text{CLE\_base}}(t,c,\text{tran},f) \tag{22}$$

Using coal as a substitution fuel and adopting the same approach as in Equations (6) and (7), we can calculate $E^{\text{CLE\_tran}}$:

$$E^{\text{CLE\_tran}}(t,c,s,f) = E_{\text{remain}}^{\text{CLE\_base}}(t,c,s,f),\ f \neq \text{coal} \tag{23}$$

$$E^{\text{CLE\_tran}}(t,c,\text{s},\text{coal}) = E_{\text{gap}}^{\text{CLE\_tran}}(t,c) \times g_s\left(E^{\text{CLE\_base}}(t,c,\text{s},\text{coal})\right) + E_{\text{remain}}^{\text{CLE\_base}}(t,c,s,\text{coal}) \tag{24}$$

In the MFR-derived scenario group, we have only one scenario **MFR_maxi**, i.e., reducing the energy gap through a 20% reduction in transportation demand and filling the remaining energy gap with renewable energy via electrification. Similar to CLE_tran (Equation (22)), the reduced energy gap $E_{\text{gap}}^{\text{MFR\_maxi}}(t,c)$ is

$$E_{\text{gap}}^{\text{MFR\_maxi}}(t,c) = E_{\text{gap}}(t,c) - 0.2 \cdot \sum_{f} E^{\text{MFR\_base}}(t,c,\text{tran},f) \tag{25}$$

Then the reduced energy gap is filled with the same approach as in MFR_rene (Equations (15)-(17)):

$$E^{\text{MFR\_maxi}}(t,c,s,f) = E_{\text{remain}}^{\text{MFR\_base}}(t,c,s,f),\ f \neq \text{elec} \tag{26}$$

$$E^{\text{MFR\_maxi}}(t,c,\text{s},\text{elec}) = E_{\text{gap}}^{\text{MFR\_maxi}}(t,c) \times g_s\left(E^{\text{MFR\_base}}(t,c,s,\text{elec})\right) + E_{\text{remain}}^{\text{MFR\_base}}(t,c,s,\text{elec}) \tag{27}$$

$$E_{\text{eg}}^{\text{MFR\_maxi}}(t,c,\text{rene}) = E_{\text{gap}}^{\text{MFR\_maxi}}(t,c) + E_{\text{eg}}^{\text{MFR\_base}}(t,c,\text{rene}) \tag{28}$$

1.2 Energy consumption for scenarios accelerating energy transition

We also evaluated the costs and benefits of different scenarios to accelerate energy transition. These scenarios are developed by achieving the renewable and electrification targets in 2050 earlier (by 5 to 20 years). The electrification targets refer to the total

electricity consumption in end-use sectors in 2050, and the renewable targets refer to the amount of renewable energy used in electricity generation in 2050.

For **MFR_base_ACyy scenarios** (yy representing the target year), we first calculate the energy consumption for the target year t_{AC}. The electricity consumption and electricity generation by renewable energy for the target year t_{AC} are the same as in 2050:

$$E_{\text{elec}}^{\text{MFR\_base\_ACyy}}(t_{\text{AC}}, c, s) = E_{\text{elec}}^{\text{MFR\_base}}(2050, c, s) \tag{29}$$

$$E_{\text{eg}}^{\text{MFR\_base\_ACyy}}(t_{\text{AC}}, c, \text{rene}) = E_{\text{eg}}^{\text{MFR\_base}}(2050, c, \text{rene}) \tag{30}$$

where $E_{\text{elec}}(t, c, s)$ is short for $E(t, c, s, \text{elec})$, and s only covers end-use sectors.

The energy consumption for other fuels is adjusted based on their original shares:

$$E^{\text{MFR\_base\_ACyy}}(t_{\text{AC}}, c, s, f) = \left(E^{\text{MFR\_base}}(t_{\text{AC}}, c, s) - E_{\text{elec}}^{\text{MFR\_base\_ACyy}}(t_{\text{AC}}, c, s)\right) \times g_f\left(E^{\text{MFR\_base}}(t_{\text{AC}}, c, \text{s}, f)\right), \tag{31}$$

$f \neq$ elec. Here, $g_f\big(E(s)\big)$ is a function to calculate the fraction of energy consumption $E(s)$ of one specific energy type in the sum of energy consumption of all energy types matching the condition specified alongside the equation ($f \neq \text{elec}$ in this case).

$$g_f\big(E(s)\big) = \frac{E(s)}{\sum E(s)} \tag{32}$$

Electricity generation by other sources other than electricity, as well as electricity generation from non-renewable sources, are adjusted across sectors and energy types based on the original shares:

$$E_{\text{eg}}^{\text{MFR\_base\_ACyy}}(t_{\text{AC}}, c, f_{\text{eg}}) = \left(E_{\text{eg}}^{\text{MFR\_base}}(t_{\text{AC}}, c) - E_{\text{eg}}^{\text{MFR\_base\_ACyy}}(t_{\text{AC}}, c, \text{rene})\right) \times g_{f_{\text{eg}}}\left(E_{\text{eg}}\big(t_{\text{AC}}, c, f_{\text{eg}}\big)\right), f_{\text{eg}} \neq \text{rene} \tag{33}$$

Based on results of 2020, 2050 and the target year, we then determine the energy consumption and electricity generation for other years through linear interpolation. For the year before t_{AC}:

$$E_{\text{elec}}^{\text{MFR\_base\_ACyy}}(t, c) = \left(E_{\text{elec}}^{\text{MFR\_base\_ACyy}}(t_{\text{AC}}, c) - E_{\text{elec}}^{\text{MFR\_base}}(2020, c)\right) \times \frac{t - 2020}{t_{\text{AC}} - 2020} + E_{\text{elec}}^{\text{MFR\_base}}(2020, c) \tag{34}$$

$$E_{\text{eg}}^{\text{MFR\_base\_ACyy}}(t, c, \text{rene}) = \left(E_{\text{eg}}^{\text{MFR\_base\_ACyy}}(t_{\text{AC}}, c, \text{rene}) - E_{\text{eg}}^{\text{MFR\_base}}(2020, c, \text{rene})\right) \times \frac{t - 2020}{t_{\text{AC}} - 2020} + E_{\text{eg}}^{\text{MFR\_base}}(2020, c, \text{rene}) \tag{35}$$

For the year after t_{AC}:

$$E_{\text{elec}}^{\text{MFR\_base\_ACyy}}(t, c)$$

$$= \left(E_{\text{elec}}^{\text{MFR\_base\_ACyy}}(t_{\text{AC}}, c) - E_{\text{elec}}^{\text{MFR\_base}}(2050, c)\right) \times \frac{2050 - t}{2050 - t_{\text{AC}}} + E_{\text{elec}}^{\text{MFR\_base}}(2050, c) \quad (36)$$

$$E_{\text{eg}}^{\text{MFR\_base\_ACyy}}(t, c, \text{rene})$$
$$= \left(E_{\text{eg}}^{\text{MFR\_base\_ACyy}}(t_{\text{AC}}, c, \text{rene}) - E_{\text{eg}}^{\text{MFR\_base}}(2050, c, \text{rene})\right) \times \frac{2050 - t}{2050 - t_{\text{AC}}} + E_{\text{eg}}^{\text{MFR\_base}}(2050, c, \text{rene}) \quad (37)$$

For **NZE_ACyy scenarios**, the scenario setup follows the same steps as in MFR_base_ACyy (Equations (29)-(37)) but starts from a different baseline NZE, which is derived from Boitier et al.(4).

Supplementary Note 2

For EU countries, energy imports from Russia exhibit both spatial and temporal variations. Spatially, overall energy dependency on Russia among EU nations ranged from 0% to 50%, with Lithuania, Slovakia, and Hungary exhibiting the highest levels of reliance. For natural gas, dependency reached as high as 95% in the Czech Republic and Latvia. An embargo on Russian energy imports would result in an average energy supply gap of 19% across the EU, with impacts ranging from 2% in Austria to 52% in Lithuania. Temporally, monthly energy consumption within the EU in 2022 was consistently and significantly lower compared to the corresponding months in 2021, particularly during the second half of the year. This reduction coincides with sharply rising energy prices. Notably, natural gas consumption falls faster than other fuels, leading to relatively higher contribution of solid fossil fuel in this period (**Fig. S6**). These observations indicate that the EU's short-term measures to the energy gap includes both "reducing energy consumption" and "increasing solid fossil fuel in the energy mix".

The total energy consumption of the EU by fuel type in selected scenarios in the short terms are shown in **Fig. S7**. Since the majority of the import from Russia is natural gas and oil, the most significant change after banning the import is the shrinking fraction of liquid and gaseous fuels, as shown in the grey and yellow bars, respectively.

Besides the changes in the energy mix, the governments also encourage the public to make efforts to reduce energy consumption from their daily life such as lowering the thermostat settings, limiting air conditioner use and preferring public transportation (5). Lowering the thermostat by 3°C (CLE_heat) leads to 3.2% reduction of total energy consumption in 2025, and reducing the private transportation demand by 20% (CLE_tran) leads to 3.4% reduction.

Fig. S7 also illustrates the total $PM_{2.5}$ emissions from the EU by fuel type in the short term. All the supply-side measures to close the energy gap would add extra $PM_{2.5}$ emissions as less clean fuels increase. The poor air pollution control measures led to relatively high emission factors in the residential sectors than other sectors such as power plants and industry (6). In consequence, the 28% of total energy consumption for residential combustion contributed 55% primary $PM_{2.5}$ emissions in the EU in 2020. Similar amplifying

effects were also confirmed by other studies (7, 8). Thus, while lowering indoor heating temperature (CLE_heat) only reduces 3% in the total energy consumption, this energy saving measure manages a 17% decrease in the total $PM_{2.5}$ emissions compared to CLE_coal. Other major air pollutants, especially the important precursors of secondary $PM_{2.5}$ such as SO_2 and NO_x, are also shown in **Fig. S7**. Unlike the primary $PM_{2.5}$, the total SO_2 emissions are dominated by coal combustion (9), as reflected by the large differences between CLE_biom and CLE_coal. As for NO_x emissions, liquid fuel consumption contributed significantly to the total emissions (10). Hence, the scenarios with coal and biomass solutions emit similar NO_x emissions because their shares of liquid fuels remained unchanged. In addition, using biofuels significantly reduces the CO_2 emissions by almost one sixth, while other scenarios accelerate global warming due to the increased coal consumption.

In the short term, all the measures to close the energy gap would increase the average $PM_{2.5}$ concentration by 23-48%. CLE_coal shows the worst average air quality with 14.0 μg/m^3 in 2025. However, it is interesting to notice that the $PM_{2.5}$ concentration of CLE_biom is only 11.2 μg/m^3 despite its highest $PM_{2.5}$ emissions. As one of the major precursors of secondary $PM_{2.5}$, SO_2 emissions from biomass fuel burning are much lower than those from coal burning (11, 12). Hence, in terms of the total ambient $PM_{2.5}$ concentration, the massive increase in biofuels would lead to a similar result with the lowered residential heating demand scenario CLE_heat. **Fig. S8** demonstrates the spatial distribution of annual $PM_{2.5}$ concentrations in 2025 for EU countries in the CLE-derived scenarios.

As reported in the Global Burden of Disease (GBD), the premature deaths attributable to $PM_{2.5}$ are over 10 times of those attributable to O_3 exposure (13). Thus, the trends of premature deaths are consistent with those of $PM_{2.5}$ exposure. Due to the substitution with more emission-intense fuels in the short term, all measures in 2025 would increase excess premature mortality by 31,000-59,000 compared to CLE_base.

Supplementary Note 3

If the energy structure and efficiency goals in 2050 can be reached by 2030 (MFR_base_AC30 scenario), the additional avoided premature mortality attributable to $PM_{2.5}$ and O_3 exposure could accumulate to 25,000 (15,600–34,300) by 2050. However, the rising costs of power plant expansion could compromise the increasing health benefits to some extent. For the entire EU, at P_2023, MFR_base_AC30 only lead to net costs of 6 (-70–110) billion EUR. In contrast, if the fuel prices rebound to the high range, MFR_base_AC30 could deliver stronger net benefits than other relatively conservative energy transition acceleration plans. Besides the health benefits from improved air quality, accelerating the energy transition also alleviates climate change by reducing additionally 678 million equivalent tons of CO_2 emissions by 2050 if the energy transition is accelerated by 20 years. At the country level, with P_2023 fuel prices, some countries such as France, Germany, Poland, Greece, Denmark and Sweden could enjoy net benefits from the accelerated energy transition (**Fig. S9**). However, not all countries will see a shift from net costs to net benefits. Due to differences in energy structures and socio-economic conditions, countries like Italy, Finland, Spain and Belgium are expected to incur net costs

regardless of fuel price variations, while France, Germany, Poland and Sweden consistently exhibit net benefits. Nevertheless, some countries do show a transition from net costs to net benefits, including Austria, Czechia, and Ireland.

For the MFR_base_AC45 and MFR_base_AC35 scenarios, cumulative premature mortality reduction due to lower $PM_{2.5}$ and O_3 exposure by 2050 are projected to be 9,400 (5,900–12,900) and 19,300 (11,900–26,500) cases, respectively. Their climate benefits, in terms of avoided CO_2 emissions, are estimated at 542 and 628 million tons by 2050. In terms of net costs, both MFR_base_AC45 and MFR_base_AC35 follow similar trends as fuel prices increase. At pre-2022 fuel prices, accelerated transitions would result in net costs of -5–24 billion EUR for MFR_base_AC45 and 10–51 billion EUR for MFR_base_AC35. As fuel prices rise, net benefits begin to emerge. At post-2022 fuel prices (including the price level P_2022), accelerated transitions would result in net benefits of 24–100 billion EUR for MFR_base_AC45 and 16–152 billion EUR for MFR_base_AC35.

Supplementary Note 4

WILIAM is a system dynamics model, developed using the software Vensim (DSS 10.1.0; https://vensim.com/). The WILIAM model is stored in its repository (https://zenodo.org/record/10813034). As shown in **Fig. S10**, within the repository, the folder "WILIAM_version_1.3" includes a user guide explaining how to run the model; the *.mdl* file containing the code that specifies how the parameters are interconnected; the folders "*model_parameters*" and "*scenario_parameters*" documenting the detailed parameters; and the folder "*Documentation&ToDo*" with technical reports that thoroughly describe the model structure, validation methods, and scenario assumptions.

Specifically, the *WILIAM_User_Guide.pdf* offers a detailed walkthrough on how to install the required software, download and open the model, run predefined scenarios, customize inputs, enable or disable specific submodules, and visualize the output results.

The *Documentation&ToDo* folder contains technical reports, which thoroughly documents the structure of the WILIAM model, validation methods, and scenario assumptions. In WILIAM, the baseline scenario is modeled based on the Long Term Strategy of the European Union (14), and the policy data is retrieved from the Climate Policy Database (15). Key targets modeled by the scenario include the EU-wide target energy consumption, reforestation, energy efficiency, renewable capacity, carbon sink capacity, and emission reduction for GHGs, with necessary adaptations to the WILIAM framework to ensure consistency.

The data sources in WILIAM are listed in its wiki page (16), under the page of each submodule.

Supplementary Note 5

Cost and benefit analysis

The difference between the additional costs and monetized benefits is referred to as the net costs of a scenario, reflecting its cost-effectiveness. That is, scenarios with higher net costs are considered less cost-effective and less preferrable, and scenarios with negative net costs are considered to save money. The net costs for scenario s are calculated by the following equation:

$$C_i^{\text{net}}(s) = C_i^{\text{ppl}}(s) + C_i^{\text{fuel}}(s) + C_i^{\text{equip}}(s) + C_i^{\text{control}}(s) + B_i^{\text{climate}}(s) + B_i^{\text{health}}(s), \qquad (38)$$

where $C_i^{\text{net}}(s)$ is the net cost for country i; $C_i^{\text{ppl}}(s)$ is the cost of power plant expansion; $C_i^{\text{fuel}}(s)$ is the cost of fuels; $C_i^{\text{equip}}(s)$ is the cost of infrastructure and equipment; $C_i^{\text{control}}(s)$ is the cost of emission control, which is derived directly from the GAINS model; $B_i^{\text{climate}}(s)$ is the benefit of climate impacts; and $B_i^{\text{health}}(s)$ is the benefit of health impacts.

The formulas for individual cost and benefit components are listed below (s is omitted for clarity).

$$C_i^{\text{ppl}} = \sum_{j \in F} \Delta\text{Cap}_{\text{i,j}} \times \text{LCOE}_{\text{i,j}} \qquad (39)$$

where $\Delta\text{Cap}_{\text{i,j}}$ is the capacity expansion for country i and fuel type j; $\text{LCOE}_{\text{i,j}}$ is the levelized cost of electricity (LCOE) for country i and fuel type j; F is the set of all fuel types.

WILIAM is applied in our LCOE calculations. WILIAM considers the spatiotemporal heterogeneity in resources by modelling region-level resource potentials exogenously (GIS-based wind/solar, EROI_{min} criteria (Energy Return on Energy Investment)) and endogenously via Land & Water module (land-use, EROI thresholds), with techno-sustainable limits (for details see descriptions in WILIAM's wiki (16)). For renewables, WILIAM parameterizes curtailment as a function of variable renewable penetration, system flexibility, and storage deployment. Internally, it emulates EnergyPLAN (for details see descriptions in EnergyPLAN (17))—an hourly-resolution energy model—to capture the relationship between influencing factors (particularly capacity expansion) and curtailment (18). This approach allows the model to dynamically approximate the expected full-load hours available for renewables, incorporating structural limits to flexibility (e.g., insufficient storage or grid upgrades).

LCOE in WILIAM is calculated using the following form:

$$\text{LCOE}(f) = \frac{\text{CAPEX}(f) + \text{OPEX}(f) \cdot \text{LT}(f) \cdot \text{FLH}(f)}{\text{LT}(f) \cdot \text{FLH}(f)} \qquad (40)$$

Here, $LCOE(f)$ refers to the LCOE values of technology f, which is calculated per year per region (EU27 is a single region in WILIAM). It is affected by:

- $CAPEX(f)$, the capital investment (USD/MW)
- $OPEX(f)$, (simplified) operation expenditure (USD/MWh), see below
- $LT(f)$, lifetime of technology (years)
- $FLH(f)$, full-load hours (h/year), equivalent to capacity factor times 8760 h/year.

CAPEX is an exogenous parameter obtained from the National Renewable Energy Laboratory (19), as in the model parameters file "energy-capacity_investment_cost.xlsx", which is available in the WILIAM's Zenodo repository (20).

OPEX represents a simplified estimate of operational expenditures in USD/MWh, calculated as follows for each technology f and region r:

$$\text{OPEX}(r;f) = \frac{1}{\eta(r;f)}\left(p_{\text{fuel}}(f) + \text{EF}(f) \cdot p_{\text{CO}_2}(r)\right) + \text{OM}(f) \qquad (41)$$

The terms in the equation are:

- $\eta(r;f)$ is the dimensionless conversion efficiency of technology f in region r
- $p_{\text{fuel}}(f)$ is the fuel cost of the technology (USD/MWh)
- $\text{EF}(f)$ is the CO_2 emission factor of technology f (tCO_2/MWh)
- $p_{\text{CO}_2}(r)$ is the CO_2 price in region r (USD/tCO_2)
- $\text{OM}(f)$ is the operation and maintenance expenditure of technology f (USD/MWh).

In the case of renewable energy, OPEX is affected only by the operation and maintenance expenditure as fuel cost and CO_2 emission factors are zero. What becomes important for renewables' LCOE is $FLH(f)$, the full-load hours of renewable technologies, through which WILIAM models the impact of curtailment (16). Specifically, curtailment is reflected in the variable "protra_max_full_load_hours_after_constraints". This variable reduces the idealized full-load hours of a technology (e.g., solar or wind) by a factor accounting for:

- Increased curtailment as renewable shares rise, especially when flexibility options lag;
- Delays in energy storage or transmission infrastructure;
- Climate-related intermittency shifts (e.g., hydropower variability);
- And policy/investment constraints affecting grid integration.

This results in an endogenous downward adjustment of renewable generation potential over time as system pressure builds. The relationship is dynamic and nonlinear, allowing feedback from energy mix choices, investment delays, and demand-side behavior.

Equipment costs associated with electrification cover electric grid infrastructure, electric industrial boilers, electric vehicle chargers, and electric heating pumps in households:

$$C_{equip_i} = \sum_j C_{ppl_i} \times r_{grid} + \sum A_{ind_i} \times P_{boiler} \times AF + \sum UCC_i \times E_{\text{elec},road,i} \times AF + \sum P_{EHP} \times A_{res_i} \times AF \quad (4[illegible]$$

wherer_{grid} is the ratio of electric grid infrastructure; A_{ind} is the electricity consumption of industry; P_{boiler} is the price of industrial electric boiler; AF is the annualization factor; UCC_i is the unit charging cost (billion EUR / PJ); $E_{\text{elec},road,i}$ is the amount of electricity consumed by road transport for country i; P_{EHP} is the average price of electric heating pumps in households; A_{res_i} is the electricity consumption in residential sectors.

The unit charging cost UCC_i is calculated in a similar fashion as Tsiropoulos et al. (21). We consider chargers with five power levels: 7kW, 22kW, 50kW, 150kW and 350kW, and categorize them into two types: slow (7kW and 22kW) and fast (50kW, 150kW and 350kW). We assume that the number of chargers at all power levels grows at the same pace. This means that the calculations for chargers within each type are identical and we only need to distinguish between the two types:

$$\mathrm{UCC} = \frac{(\mathrm{UCC_{slow}} \times E_{\mathrm{slow}} + \mathrm{UCC_{fast}} \times E_{\mathrm{fast}})}{E_{\mathrm{slow}} + E_{\mathrm{fast}}} \tag{43}$$

where $E_{\mathrm{elec},road} = E_{\mathrm{slow}} + E_{\mathrm{fast}}$ and subscripts i, elec, road are dropped for brevity. As capacity expansion promotes technology advancements, we adopt learning rates $r_{\mathrm{capex,slow}}^{\mathrm{learn}}$, $r_{\mathrm{capex,fast}}^{\mathrm{learn}}$, and $r_{\mathrm{install}}^{\mathrm{learn}}$ that respectively indicate how much the capital expenses for slow and fast chargers, as well as installation costs for both charger types, drop when the number of a certain type of charger doubles. which means that the per-charger cost CC at a target year can be adjusted according to the change in road vehicle electricity consumption between target and baseline years (E/E_0 in the equation):

$$\mathrm{CC_{slow}} = \mathrm{CAPEX_{slow}} \times {r_{\mathrm{capex,slow}}^{\mathrm{learn}}}^{E/E_0} + \mathrm{INSTALL_{slow}} \times {r_{\mathrm{install}}^{\mathrm{learn}}}^{E/E_0} \tag{44}$$

and $\mathrm{CC_{fast}}$ is calculated analogously. Here, the values on the right-hand side are from Tsiropoulos et al. (21) except for the electricity consumption which is from our model. The per-charger cost can be converted to per-unit-electricity cost with the charger's power and annual run time (ART):

$$\mathrm{UCC_{slow}} = \frac{\mathrm{CC_{slow}}}{\mathrm{P_{slow}} \times \mathrm{ART}} \tag{45}$$

And the annualization factor AF is calculated through

$$AF = \frac{r}{1-(1+r)^L} \tag{46}$$

where r is the discount rate, which is set to 7%; L is the lifetime of various equipment, which is set to 15 years for electric vehicle chargers, 20 years for industry electric equipment, 15 years for residential electric equipment.

$$C_i^{\mathrm{fuel}} = \sum_{j \in F} A_{i,j}^{\mathrm{fuel}} \times P_{i,j}^{\mathrm{fuel}} \tag{47}$$

where $A_{i,j}^{\mathrm{fuel}}$ is the fuel consumption for country i and fuel type j; $P_{i,j}^{\mathrm{fuel}}$ is the fuel price for country i and fuel type j.

$$B_i^{\mathrm{climate}} = \sum_{y \in Y} \mathrm{SCC_y} \times E_{i,y}^{\mathrm{CO_2}} \tag{48}$$

where $\mathrm{SCC_y}$ is the social cost of carbon dioxide derived from literature (22); and $E_{i,y}^{\mathrm{CO_2}}$ is the CO_2 emission for country i and year y; Y represents all years modeled in the study.

$$B_i^{\text{health}} = \sum_{y \in Y} M_{i,y} \times \text{VSL}_{\text{i,y}} \quad (49)$$

where $M_{i,y}$ is the premature mortality induced by $PM_{2.5}$ and O_3 exposure for country i and year y; $\text{VSL}_{\text{i,y}}$ is the value of a statistical life for country i and year y, computed as

$$\text{VSL}_{\text{i,y}} = \text{VSL}_{\text{base}} \times \left(\frac{I_{i,y}}{I_{\text{base}}}\right)^{\epsilon} \quad (50)$$

where VSL_{base} is the VSL in the base year; $I_{i,y}$ is the GDP per capita for country i and year y; I_{base} is the GDP per capita in the base year; ϵ is the elasticity. ϵ is set to 0.5 in this study. The parameter values are listed in the data repository.

Supplementary Note 6

Sensitivity analysis

Fig. S11 illustrates the sensitivity analysis of net costs of accelerating energy transition in the EU under P_2022. In the sensitivity analysis, net costs remain negative across all four acceleration scenarios (MFR_base_AC45, MFR_base_AC40, MFR_base_AC35, MFR_base_AC30) and for every parameter variation. For the levelized cost of electricity (LCOE), lowering the value to 50% increased the total net costs by 33–69 billion EUR, whereas increasing it to 150% reduced the costs by 32–69 billion EUR. For the discount rate of equipment costs, reducing it to 4% decreased the total net costs by 6–26 billion EUR, whereas increasing it to 10% increased the net costs by 7–29 billion EUR. For the elasticity factor of VSL, decreasing it to 0.3 decreased the net costs by 0 to –2 billion EUR, whereas increasing it to 1 increased the costs by 1–3 billion EUR. For the social cost of CO_2 (SC-CO_2), lowering the discount rate to 1.5% reduced the net costs by 54–68 billion EUR, while raising it to 3% increased the costs by 51–63 billion EUR. For the emission control cost, reducing the discount rate to 4% changed the total net costs by -4–7 billion EUR, whereas increasing discount rate to 20% changed the costs by 6–(-4) billion EUR. For the value of a statistical life (VSL), lowering the value to 50% increased the total net costs by 29–70 billion EUR, whereas increasing it to 150% reduced the costs by 24–64 billion EUR.

Supplementary Note 7

Robustness test on endogenous fuel price responses

We performed a robustness analysis to examine how endogenous fuel prices respond to alternative EU-side demand and supply strategies in WILIAM v1.3 with the Russian-import-halt extension (WILIAM-RIH). We first introduced a switch within WILIAM to represent halting the Russian imports for EU countries. We then established a set of scenarios by modifying three key aspects in WILIAM: final energy substitution strategies and final energy substitution rates in non-energy sectors, and capacity expansion priorities in energy sectors. The baseline case (B0_baseline) follows an NDC-consistent setup, with

parameter files listed in our repository (see Supplementary Note 4). Eight perturbation scenarios were evaluated (**Table S2**) in two families. The first family perturbs only substitution rates while keeping capacity-expansion priorities at baseline (SR_coal_mod, SR_coal_str, SR_elec_mod, SR_elec_str). The second family perturbs only capacity-expansion priorities while keeping final energy substitution at baseline (CE_coal_mod, CE_coal_str, CE_elec_mod, CE_elec_str). In the capacity-expansion family, the strong cases use the full fossil/uranium-oriented or renewable-oriented priority vectors, and the moderate cases use midpoint vectors between the corresponding strong vector and current-trend priorities. The moderate perturbation (+0.1) represents a measurable but limited acceleration of substitution, while the high perturbation (+0.5) represents a stress-test level that forces rapid reallocation of final energy use. The augmented model WILIAM-RIH and all scenarios' parameter files are uploaded in the data repository.

All results are reported as deviations from the baseline scenario. For **Fig. S12**, we first applied a trailing five-year moving average to both scenario and baseline prices, and then calculated the relative change. Non-EU substitution inputs were kept at baseline so that the tested signal is centered on EU strategy design. The three-panel comparison (A: coal, B: oil, C: gas) shows a consistent pattern: coal prices increase under fossil-oriented capacity expansion and decrease under renewable-oriented expansion; gas prices are generally lower than baseline, with the deepest reductions in renewable-oriented expansion cases; oil prices show mixed responses, with strong positive deviations in some substitution-rate stress cases and negative deviations in renewable-oriented expansion cases. Across all scenarios, the observed ranges are approximately −36.8% to +12.6% for coal, −10.9% to +4.2% for gas, and −40.2% to +28.0% for oil. These fuel-price envelopes were then propagated to the net-cost robustness comparisons (**Figs. S13-S16**), using CE_elec_str and CE_coal_str as upper and lower bounds from the scenario set. Overall, the endogenous fuel-price responses remain bounded and do not change the ranking of pathways or the main conclusions.

Figures

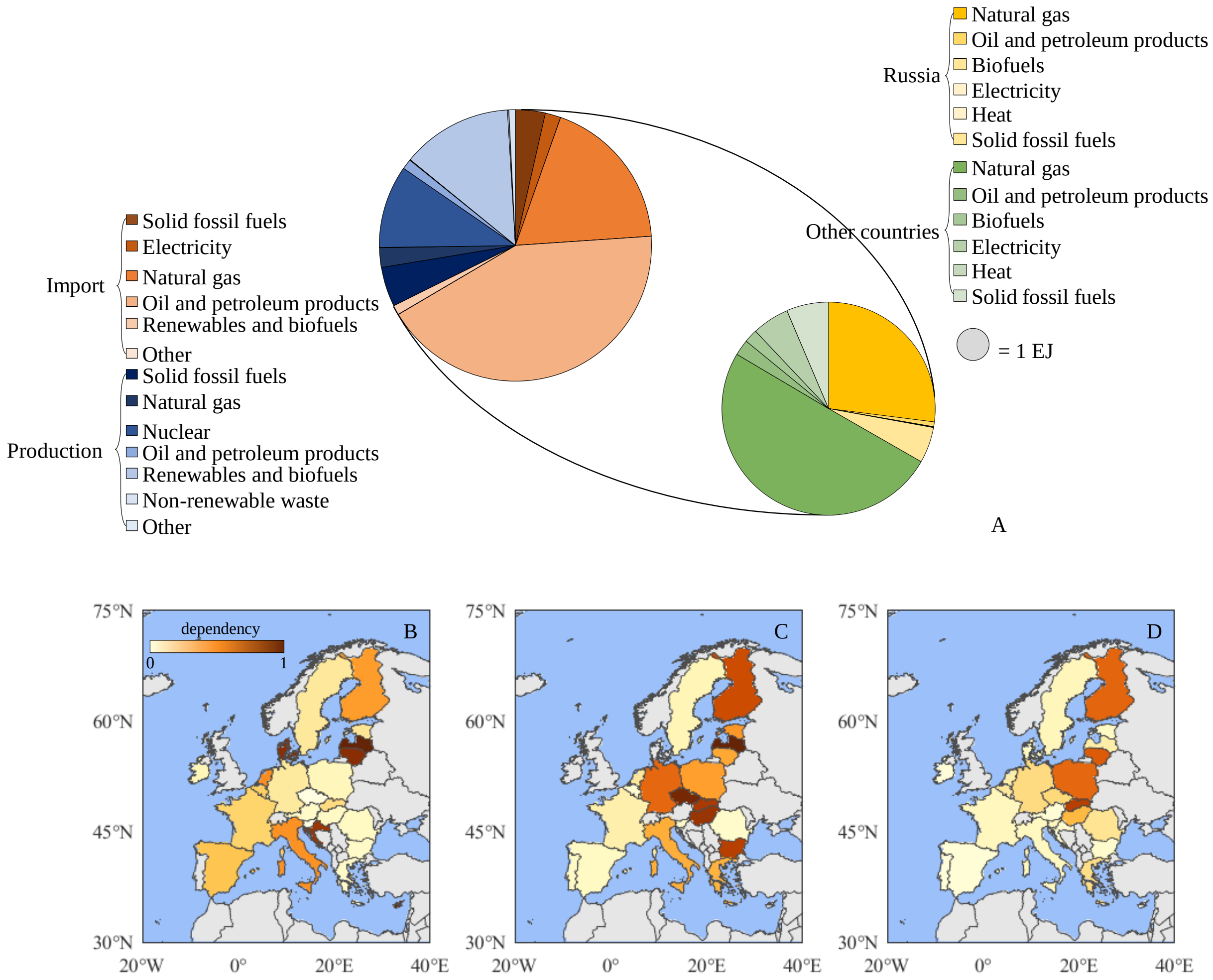

Fig. S1. EU energy dependency in 2020. (A) Composition of total energy supply (on a primary energy basis) and the share contributed by energy imports. In the pie chart, slice areas represent each source's share of total supply. (B–D) Country-level dependency on Russia for solid fossil fuels (B), natural gas (C), and oil & petroleum products (D). Data source: Eurostat, 2023. Note that, in (A), imported electricity from non-EU countries is also considered as part of the energy supply while domestic electricity is not considered.

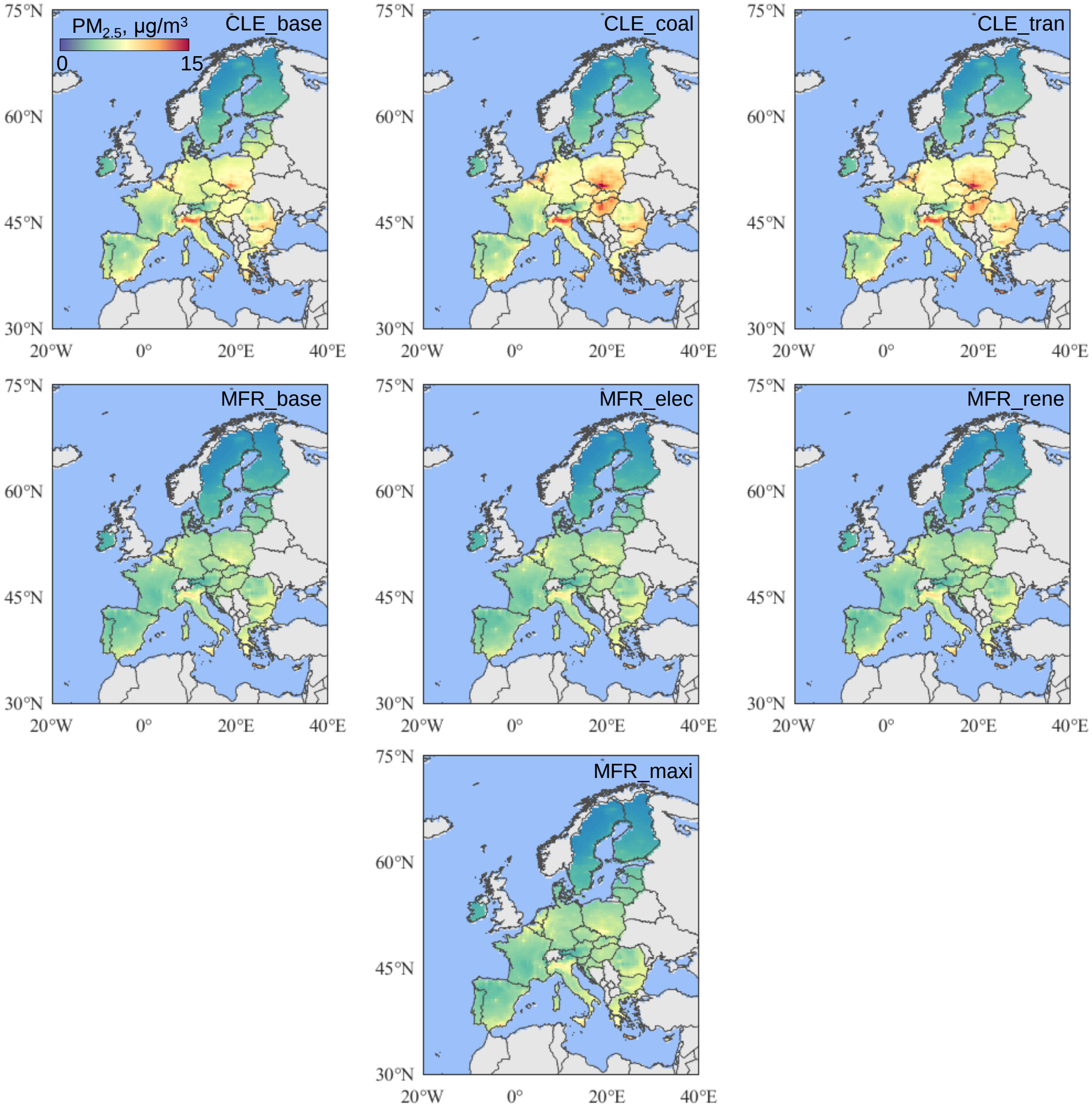

Fig. S2 Spatial distribution of simulated $PM_{2.5}$ concentrations across EU countries in 2050 under different scenarios. The maps show the simulated annual average $PM_{2.5}$ concentrations (μg/m^3) across Europe from GAINS for seven scenarios: CLE_base, CLE_coal, CLE_tran, MFR_base, MFR_elec, MFR_rene, and MFR_maxi. The color scale is consistent across all panels.

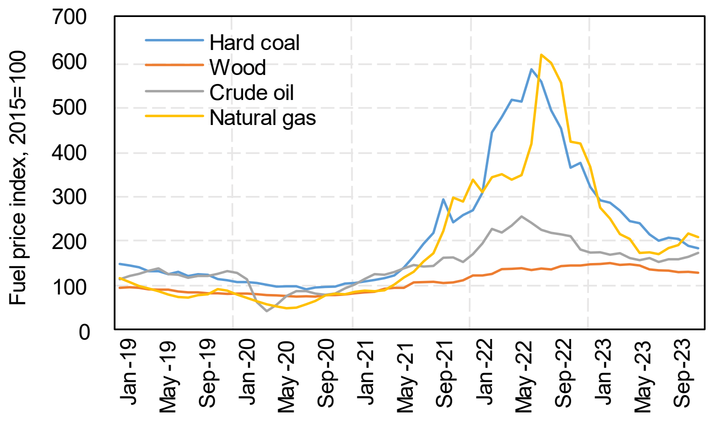

Fig. S3 Monthly import price index of major fuels in Germany from 2019 to 2023. The figure shows the trends in import price indices (2015 = 100) for natural gas, hard coal, crude oil, and mineral oil products over the period 2019–2023. Data reflect monthly fluctuations. Data source: Statistisches Bundesamt, 2024.

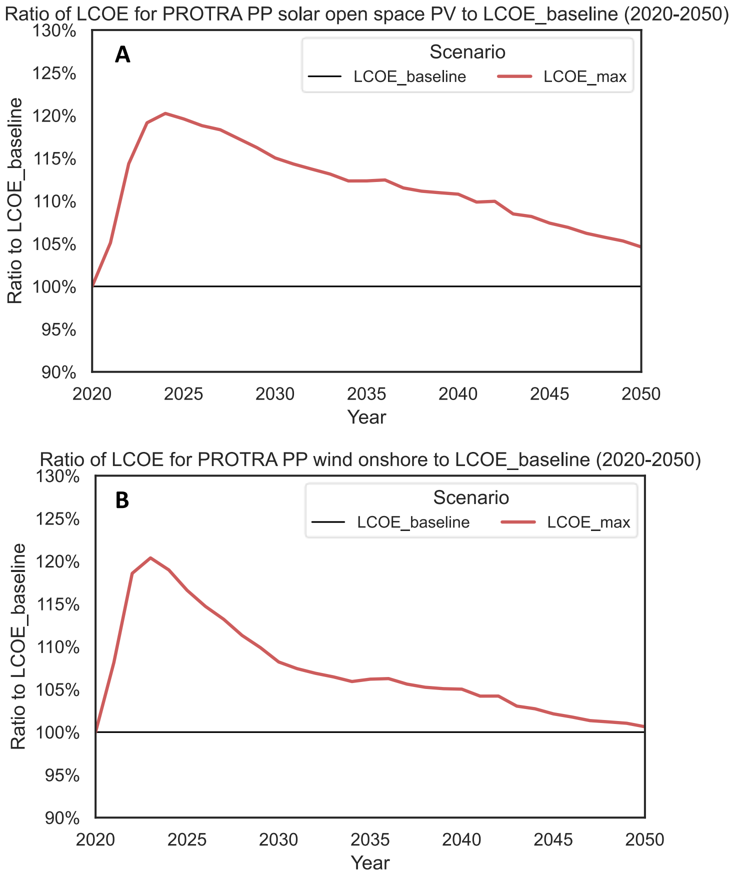

Fig. S4 Levelized costs of electricity (LCOE) trends for power plants (PP) solar open space photovoltaic (PV) (A) and wind onshore (B) from 2020 to 2050 in the EU. LCOE_baseline follows capacity expansion consistent with each country's nationally

determined contribution under the Paris Agreement. LCOE_max assigns the highest possible expansion priorities for renewable energies, though their expansion is still constrained by the potential.

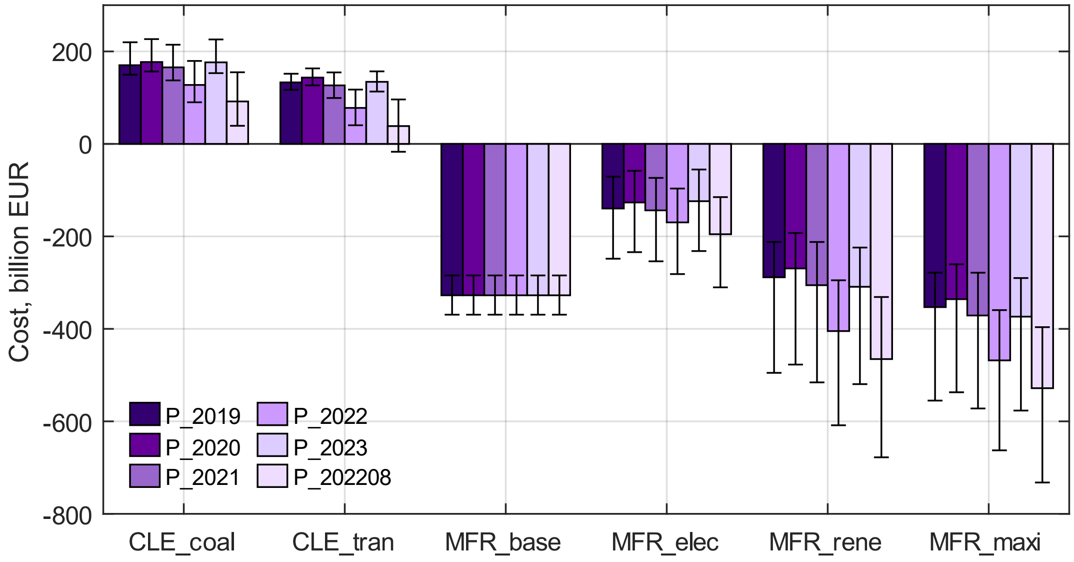

Fig. S5 Net costs different scenarios for the EU in 2050 under six fuel price levels. The bars show the net costs of different scenarios relative to the CLE_base scenario, representing the difference in additional costs (in billion EUR) between each scenario and CLE_base. Results are presented for six fuel price levels (P_2019, P_2020, P_2021, P_2022, P_2023, and P_202208). Error bars indicate the 95% confidence intervals (CI).

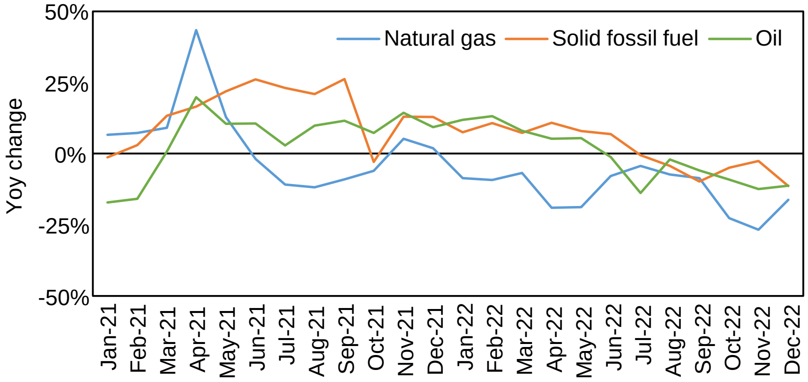

Fig. S6 The monthly changes of energy consumption in the EU in 2021 and 2022. "Yoy change" represents year over year change per month.

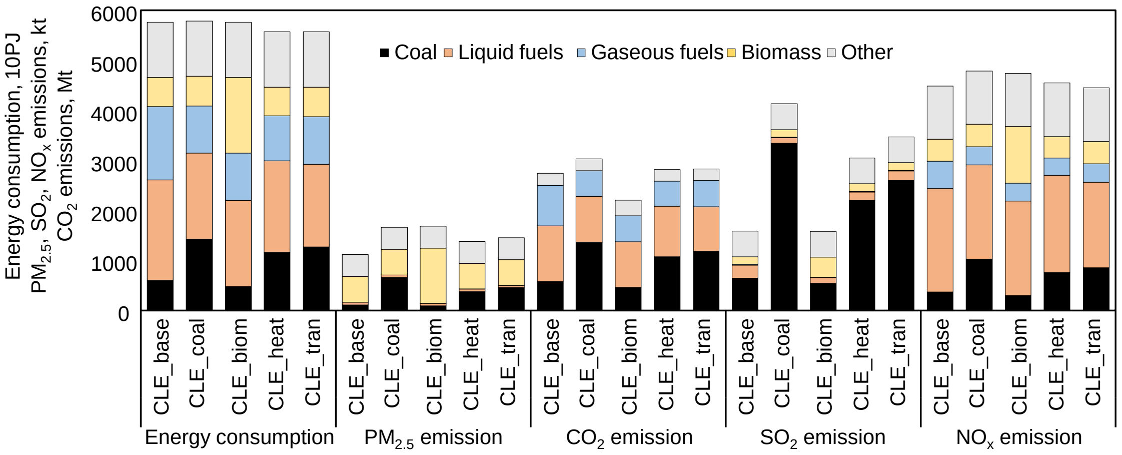

Fig. S7 Energy consumption and pollutant emissions associated with measures to close the energy gap in the EU in 2025. The energy consumption, $PM_{2.5}$, CO_2, SO_2, and NO_x emissions under different scenarios are showed in stacked bars. Results are disaggregated by fuel type: coal, liquid fuels, gaseous fuels, biomass, and other sources. Scenarios include CLE_base, CLE_coal, CLE_biom, CLE_heat, and CLE_tran.

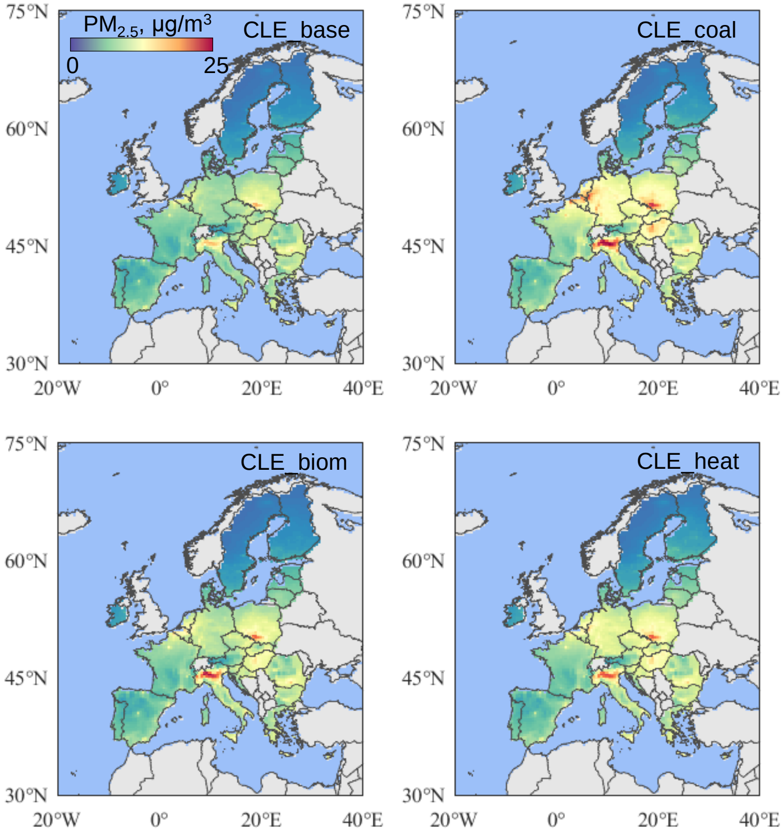

Fig. S8 Spatial distribution of simulated $PM_{2.5}$ concentrations across EU countries in 2025 under different scenarios. The maps show the simulated annual average $PM_{2.5}$

concentrations (μg/m^3) across Europe in 2025 for four scenarios: CLE_base, CLE_coal, CLE_biom, and CLE_heat. The color scale is consistent across all panels.

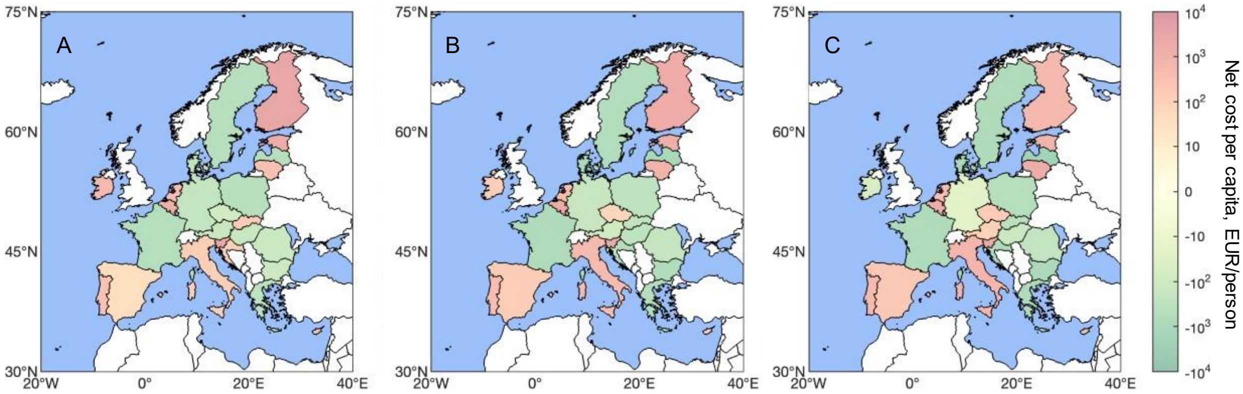

Fig. S9 Cumulative net cost per capita in EU countries by 2050 under the fuel price level P_2023. The maps display the country-level cumulative net cost per capita (EUR/person) from 2020 to 2050 under three scenarios: (A) MFR_base_AC45, (B) MFR_base_AC35, and (C) MFR_base_AC30. Colors represent the magnitude and sign of the net costs on a logarithmic scale, with green indicating net savings and red indicating net costs.

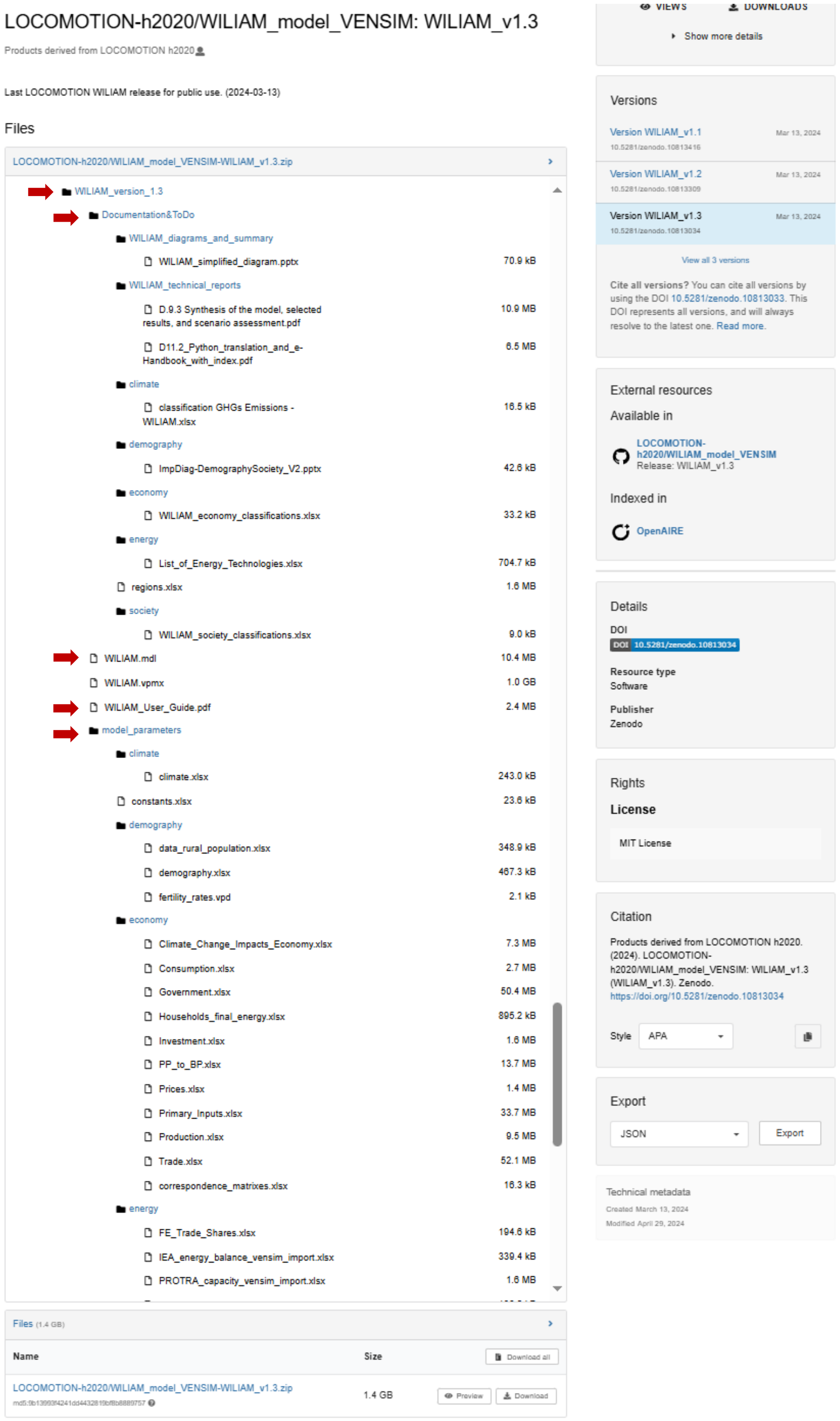

Fig. S10 Screenshot of WILIAM repository. Red arrows highlight the relevant files and folders mentioned in Supplementary Note 4.

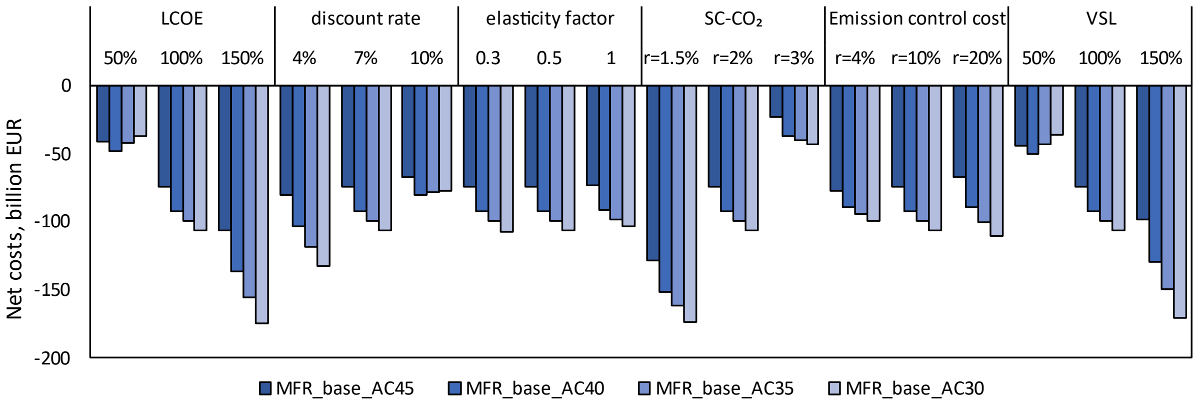

Fig. S11 Sensitivity analysis of net costs for accelerating the EU energy transition. Four scenarios MFR_base_AC45, MFR_base_AC40, MFR_base_AC35, MFR_base_AC30 under price level P_2022 are examined across six input parameters: levelized cost of electricity (LCOE: 50 %, 100 %, 150 %), discount rate (4 %, 7 %, 10 %), elasticity factor (0.3, 0.5, 1.0), social cost of CO_2 (discount rates 1.5 %, 2 %, 3 %), emission-control interest cost (4 %, 10 %, 20 %), and value of statistical life (VSL: 50 %, 100 %, 150 %).

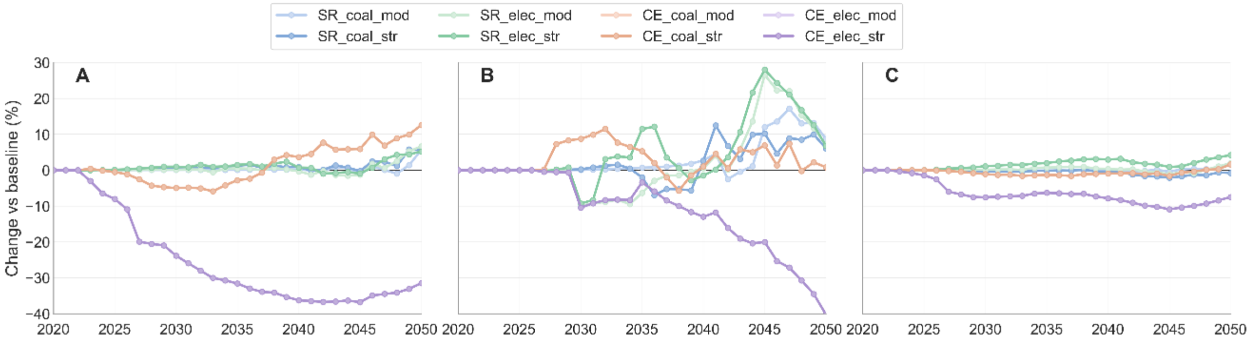

Fig. S12. Fuel price responses relative to baseline under robustness scenarios. Panel A shows coal prices, panel B shows oil prices, and panel C shows gas prices. For each scenario, the series is shown in relative change to the baseline. Scenarios include substitution-rate perturbations only (SR_coal_mod, SR_coal_str, SR_elec_mod, SR_elec_str) and capacity-expansion-priority perturbations only (CE_coal_mod, CE_coal_str, CE_elec_mod, CE_elec_str). All scenarios are compared to the common baseline (B0_baseline). In all groups, mod denotes a moderate perturbation and str denotes a strong, stress-test perturbation.

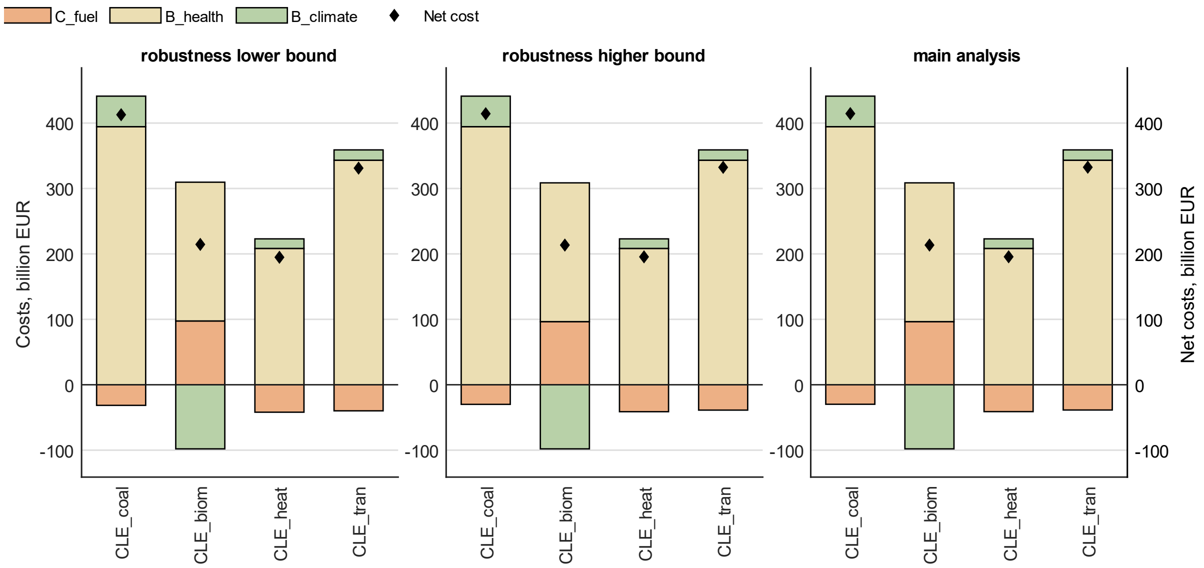

Fig. S13 Decomposition of net costs in 2025 for the EU (robustness test). The two left panels show the net cost decomposition with coal price at the robustness test's lower and higher bound, respectively; the figure from our main analysis is reproduced as the right panel for comparison. For each scenario (CLE_coal, CLE_biom, CLE_heat, CLE_tran), stacked bars report C_fuel, B_health, and B_climate (billion EUR, left y-axis), while black diamonds indicate total net cost (right y-axis). All values shown are relative to CLE_base.

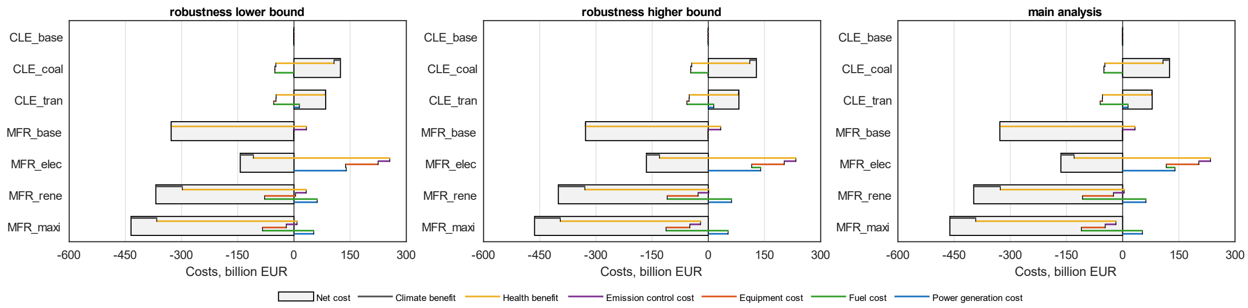

Fig. S14 Decomposition of net costs in 2050 for the EU (robustness test). The two left panels show the net cost decomposition with coal price at the robustness test's lower (CE_elec_str) and higher bound (CE_coal_str), respectively; the figure from the main analysis is reproduced as the right panel for comparison. For each scenario (MFR_maxi to CLE_base), the gray bar indicates net cost (billion EUR). Components include C_ppl (power plant investment), C_fuel (fuel costs), C_equip (infrastructure costs), C_emis (emission control costs), B_health (monetized health benefits), and B_climate (monetized climate benefits).

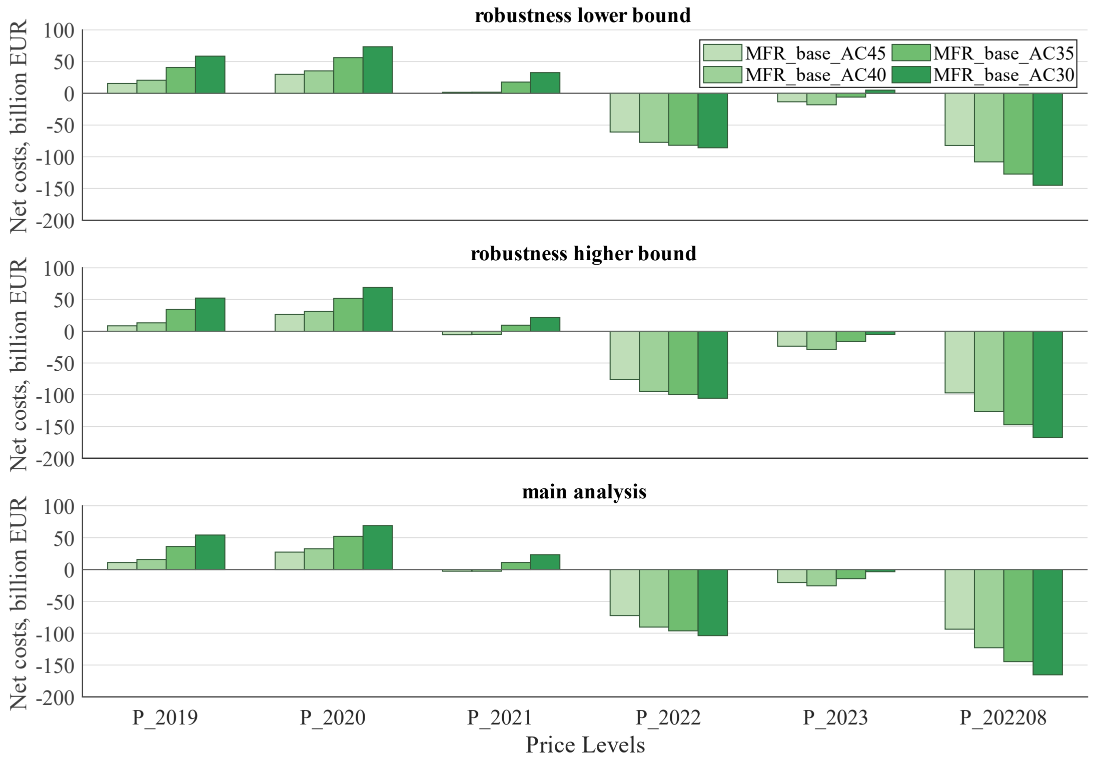

Fig. S15 Net costs for accelerating the EU energy transition during the period of 2020 to 2050 under MFR pathway (robustness test). The two top panels show the net costs using coal price at the robustness test's lower (CE_elec_str) and higher bound (CE_coal_str), respectively; the figure from the main analysis is reproduced as the bottom panel for comparison. Each panel shows the EU cumulative net costs (billion EUR) from 2020 to 2050 for scenarios MFR_base_AC45, MFR_base_AC40, MFR_base_AC35 and MFR_base_AC30 at six different price levels.

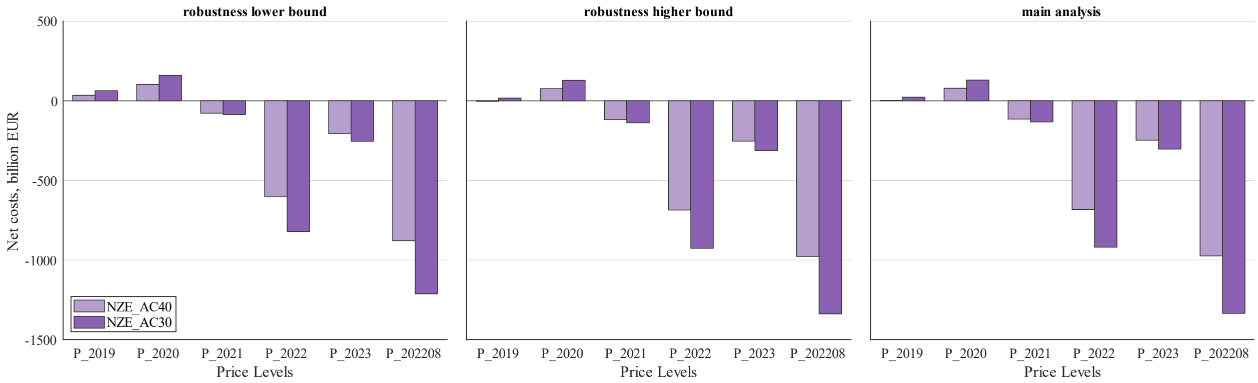

Fig. S16 Net costs for accelerating the EU energy transition during the period of 2020 to 2050 under NZE pathway (robustness test). The two left panels show the net cost decomposition with coal price at the robustness test's lower (CE_elec_str) and higher bound (CE_coal_str), respectively; the figure from the main analysis is reproduced as the

right panel for comparison. Each panel shows the EU cumulative net costs (billion EUR) from 2020 to 2050 for scenarios NZE_AC40 and NZE_AC30 at different price levels.

Tables

Table S1 Detailed configuration settings and input files for the GAINS simulations, specifying sectoral data sources, file formats, and scenario-specific parameters.

	**Configuration & input files**	**Sources**
Energy, mobile and process sectors	'Energy_total_[scenario name]_[specific year].xlsx', e.g., ‘Energy_total_CLE_2050.xlsx’, country-level activity pathway and scenario-specific inputs	Zenodo repository: 10.5281/zenodo.16738861
Agriculture	'CURRENT_PATTERN_2019_AGR', baseline agricultural activity pattern calibrated to 2019	GAINS provided[1]
Fluorinated greenhouse gases (FGAS) sources	'ETP_6DS_2018_FGAS', activity trajectory aligned with the IEA Energy Technology Perspectives 6°C Scenario, 2018 edition	GAINS provided[1]
Volatile organic carbons (VOC) sources	'CURRENT_PATTERN', default solvent and other VOC-related activity pathway	GAINS provided[1]
Control strategies	'WHOL_CLE17Fv6', country-specific control strategies for CLE; 'WHOLMFR_15717_27', country-specific control strategies for MFR	Same as in the 'ECLIPSE_V6b_CLE_base' and 'ECLIPSE_V6b_MFR_base', GAINS provided[1]

[1] https://gains.iiasa.ac.at/models/

Table S2. Scenario definitions for the robustness analysis of endogenous energy price responses under two perturbation families relative to the NDC-consistent baseline (B0_baseline): substitution rate perturbations (SR) and capacity expansion priority perturbations (CE). The SR scenarios (SR_coal_mod, SR_coal_str, SR_elec_mod, SR_elec_str) vary substitution direction (coal- vs electricity-directed) and perturbation intensity (moderate vs strong) while keeping expansion priorities at baseline. The CE scenarios (CE_coal_mod, CE_coal_str, CE_elec_mod, CE_elec_str) vary expansion direction (fossil/uranium-oriented vs renewable-oriented) and perturbation intensity (moderate vs strong) while keeping final energy substitution at baseline. Moderate CE cases are defined as midpoint vectors between current-trend priorities and the corresponding strong-direction priority vectors.

Scenario ID	Description
B0_baseline	Baseline reference trajectory for all relative comparisons.
SR_coal_mod	Substitution-rate perturbation only: coal-directed, moderate level, with expansion priorities kept at baseline.
SR_coal_str	Substitution-rate perturbation only: coal-directed, strong level, with expansion priorities kept at baseline.
SR_elec_mod	Substitution-rate perturbation only: electricity-directed, moderate level, with expansion priorities kept at baseline.
SR_elec_str	Substitution-rate perturbation only: electricity-directed, strong level, with expansion priorities kept at baseline.
CE_coal_mod	Capacity-expansion perturbation only: fossil/uranium-oriented direction, moderate level (midpoint between current-trend and fossil-oriented priorities), with final-energy substitution kept at baseline.
CE_coal_str	Capacity-expansion perturbation only: fossil/uranium-oriented direction, strong level (fossil-oriented priority set), with final-energy substitution kept at baseline.
CE_elec_mod	Capacity-expansion perturbation only: renewable-oriented direction, moderate level (midpoint between current-trend and renewable-oriented priorities), with final-energy substitution kept at baseline.
CE_elec_str	Capacity-expansion perturbation only: renewable-oriented direction, strong level (renewable-oriented priority set), with final-energy substitution kept at baseline.

SI References

1. International Institute for Applied Systems Analysis. Global Emission Fields of Air Pollutants and GHG. https://iiasa.ac.at/models-tools-data/global-emission-fields-of-air-pollutants-and-ghgs. Accessed May 12, 2025.
2. International Institute for Applied Systems Analysis (IIASA). Greenhouse Gas - Air Pollution Interactions and Synergies (GAINS), https://gains.iiasa.ac.at/models/.

3. Eurostat (2025) Cooling and heating degree days by country – annual data. Eurostat, Luxembourg. Available at: https://ec.europa.eu/eurostat/databrowser/view/nrg_chddr2_a/default/table.
4. Boitier B*, et al.* (2023) A multi-model analysis of the EU's path to net zero. *Joule* 7(12):2760-2782.
5. European Commission and International Energy Agency, *Playing My Part* (2022). https://www.ie-a.org/reports/playing-my-part.
6. Huang Y*, et al.* (2014) Quantification of Global Primary Emissions of $PM_{2.5}$, PM_{10}, and TSP from Combustion and Industrial Process Sources. *Environmental Science & Technology* 48(23):13834-13843.
7. Yun X*, et al.* (2020) Residential solid fuel emissions contribute significantly to air pollution and associated health impacts in China. *Science Advances* 6(44):eaba7621.
8. Meng W*, et al.* (2021) Synergistic Health Benefits of Household Stove Upgrading and Energy Switching in Rural China. *Environmental Science & Technology* 55(21):14567-14575.
9. Smith SJ*, et al.* (2011) Anthropogenic sulfur dioxide emissions: 1850–2005. *Atmos. Chem. Phys.* 11(3):1101-1116.
10. Huang T*, et al.* (2017) Spatial and Temporal Trends in Global Emissions of Nitrogen Oxides from 1960 to 2014. *Environmental Science & Technology* 51(14):7992-8000.
11. Zhong Q*, et al.* (2018) Distinguishing Emission-Associated Ambient Air $PM_{2.5}$ Concentrations and Meteorological Factor-Induced Fluctuations. *Environmental Science & Technology* 52(18):10416-10425.
12. Yun X*, et al.* (2021) Coal Is Dirty, but Where It Is Burned Especially Matters. *Environmental Science & Technology* 55(11):7316-7326.
13. GBD 2019 Risk Factors Collaborators (2020) Global burden of 369 diseases and injuries in 204 countries and territories, 1990-2019: a systematic analysis for the Global Burden of Disease Study 2019. *The Lancet* 396(10258):1204-1222.
14. European Commission (2020) 2050 long-term strategy. in *Directorate-General for Climate Action*.
15. NewClimate Institute, Wageningen University and Research & PBL Netherlands Environmental Assessment Agency. (2023). Climate Policy Database. DOI: 10.5281/zenodo.10869734.
16. LOCOMOTION-h2020/WILIAM_model_VENSIM wiki (2025) Available at: https://github.com/LOCOMOTION-h2020/WILIAM_model_VENSIM/wiki. Accessed Jul 14 2025.
17. EnergyPLAN Documentation (2024), EnergyPLAN / Department of Development and Planning, Aalborg University. Available at: https://energyplan.eu/about-energyplan/documentation/.
18. Parrado-Hernando G, Herc L, Feijoo Palacios FA, & Capellán-Pérez I (2024) Capturing Features of Hourly-resolution Energy Models in an Integrated Assessment Model: an application to the Europe-27 region. *Energy*:131903.
19. National Renewable Energy L (2023) 2023 Electricity ATB Technologies. (National Renewable Energy Laboratory, Golden, CO).
20. LOCOMOTION Consortium, *WILIAM Model v1.3* (2024). https://doi.org/10.5281/zenodo.10813034.
21. Tsiropoulos I, Siskos P, & Capros P (2022) The cost of recharging infrastructure for electric vehicles in the EU in a climate neutrality context: Factors influencing investments in 2030 and 2050. *Applied Energy* 322:119446.
22. Rennert K*, et al.* (2022) Comprehensive evidence implies a higher social cost of CO_2. *Nature* 610(7933):687-692.
